# A Cost-effectiveness Analysis of Multigene Testing for All Patients With Breast Cancer

**DOI:** 10.1001/jamaoncol.2019.3323

**Published:** 2019-10-03

**Authors:** Li Sun, Adam Brentnall, Shreeya Patel, Diana S. M. Buist, Erin J. A. Bowles, D. Gareth R. Evans, Diana Eccles, John Hopper, Shuai Li, Melissa Southey, Stephen Duffy, Jack Cuzick, Isabel dos Santos Silva, Alec Miners, Zia Sadique, Li Yang, Rosa Legood, Ranjit Manchanda

**Affiliations:** 1 Department of Health Services Research and Policy, London School of Hygiene & Tropical Medicine, London, United Kingdom; 2 Centre for Experimental Cancer Medicine, Barts Cancer Institute, Queen Mary University of London, London, United Kingdom; 3 Centre for Cancer Prevention, Wolfson Institute of Preventive Medicine, Queen Mary University of London, London, United Kingdom; 4 Kaiser Permanente Washington Health Research Institute, Seattle, Washington; 5 Genomic Medicine, Manchester Academic Health Science Centre, Manchester Universities Foundation Trust, St Mary’s Hospital, The University of Manchester, Manchester, United Kingdom; 6 Cancer Sciences Academic Unit, Faculty of Medicine and Cancer Sciences, University of Southampton, Southampton, United Kingdom; 7 Centre for Epidemiology and Biostatistics, Melbourne School of Population and Global Health, Faculty of Medicine, Dentistry and Health Sciences, University of Melbourne, Melbourne, Australia; 8 Precision Medicine, School of Clinical Sciences at Monash Health, Monash University, Clayton, Australia; 9 Department of Clinical Pathology, Melbourne Medical School, Melbourne University, Melbourne, Australia; 10 Cancer Epidemiology Division, Cancer Council Victoria, Victoria, Australia; 11 Department of Noncommunicable Disease Epidemiology, London School of Hygiene & Tropical Medicine, London, United Kingdom; 12 School of Public Health, Peking University, Beijing, China; 13 Department of Gynaecological Oncology, Barts Health National Health System Trust, Royal London Hospital, London, United Kingdom; 14 MRC (Medical Research Counsel) Clinical Trials Unit at UCL, Institute of Clinical Trials and Methodology, Faculty of Population Health Sciences, University College London, London, United Kingdom

## Abstract

**Importance:**

Moving to multigene testing for all women with breast cancer (BC) could identify many more mutation carriers who can benefit from precision prevention. However, the cost-effectiveness of this approach remains unaddressed.

**Objective:**

To estimate incremental lifetime effects, costs, and cost-effectiveness of multigene testing of all patients with BC compared with the current practice of genetic testing (*BRCA*) based on family history (FH) or clinical criteria.

**Design, Setting, and Participants:**

This cost-effectiveness microsimulation modeling study compared lifetime costs and effects of high-risk *BRCA1/BRCA2/PALB2* (multigene) testing of all unselected patients with BC (strategy A) with *BRCA1/BRCA2* testing based on FH or clinical criteria (strategy B) in United Kingdom (UK) and US populations. Data were obtained from 11 836 patients in population-based BC cohorts (regardless of FH) recruited to 4 large research studies. Data were collected and analyzed from January 1, 2018, through June 8, 2019. The time horizon is lifetime. Payer and societal perspectives are presented. Probabilistic and 1-way sensitivity analyses evaluate model uncertainty.

**Interventions:**

In strategy A, all women with BC underwent *BRCA1/BRCA2/PALB2* testing. In strategy B, only women with BC fulfilling FH or clinical criteria underwent *BRCA* testing. Affected *BRCA/PALB2* carriers could undertake contralateral preventive mastectomy; *BRCA* carriers could choose risk-reducing salpingo-oophorectomy (RRSO). Relatives of mutation carriers underwent cascade testing. Unaffected relative carriers could undergo magnetic resonance imaging or mammography screening, chemoprevention, or risk-reducing mastectomy for BC risk and RRSO for ovarian cancer (OC) risk.

**Main Outcomes and Measures:**

Incremental cost-effectiveness ratio (ICER) was calculated as incremental cost per quality-adjusted life-year (QALY) gained and compared with standard £30 000/QALY and $100 000/QALY UK and US thresholds, respectively. Incidence of OC, BC, excess deaths due to heart disease, and the overall population effects were estimated.

**Results:**

*BRCA1/BRCA2/PALB2* multigene testing for all patients detected with BC annually would cost £10 464/QALY (payer perspective) or £7216/QALY (societal perspective) in the United Kingdom or $65 661/QALY (payer perspective) or $61 618/QALY (societal perspective) in the United States compared with current *BRCA* testing based on clinical criteria or FH. This is well below UK and US cost-effectiveness thresholds. In probabilistic sensitivity analysis, unselected multigene testing remained cost-effective for 98% to 99% of UK and 64% to 68% of US health system simulations. One year’s unselected multigene testing could prevent 2101 cases of BC and OC and 633 deaths in the United Kingdom and 9733 cases of BC and OC and 2406 deaths in the United States. Correspondingly, 8 excess deaths due to heart disease occurred in the United Kingdom and 35 in the United States annually.

**Conclusions and Relevance:**

This study found unselected, high-risk multigene testing for all patients with BC to be extremely cost-effective compared with testing based on FH or clinical criteria for UK and US health systems. These findings support changing current policy to expand genetic testing to all women with BC.

## Key Points

**Question:**

Is unselected genetic testing of all women with breast cancer cost-effective compared with testing based on clinical criteria or family history?

**Findings:**

In this cost-effectiveness microsimulation modeling study incorporating data from 11 836 women, unselected *BRCA1/BRCA2/PALB2* testing at breast cancer diagnosis was extremely cost-effective compared with *BRCA1/BRCA2* testing based on clinical criteria or family history for UK and US health systems, with incremental cost-effectiveness ratios of £10 464 or £7216 and $65 661 or $61 618 per quality-adjusted life-year, respectively. One year’s unselected panel genetic testing could prevent 2101 cases of breast or ovarian cancer and 633 deaths in the United Kingdom and 9733 cases and 2406 deaths in the United States.

**Meaning:**

These findings support changing current policy to expand genetic testing to all women with breast cancer.

## Teaser

This cost-effectiveness microsimulation modeling study estimates incremental lifetime effects, costs, and cost-effectiveness of multigene testing of all patients with breast cancer compared with selective testing based on family history or clinical criteria.

## Introduction

Current national and international guidelines recommend genetic testing in women with breast cancer (BC) who fulfill recognized or established family history (FH) or clinical criteria. These criteria are surrogates for *BRCA* (*BRCA1* [OMIM 113705] and *BRCA2* [OMIM 600185]) probability, with genetic testing usually offered at approximately a 10% probability threshold of being a *BRCA* carrier.^1,2^ Being a *BRCA* (mutation) carrier refers to carrying an inheritable genetic pathogenic variant that predisposes to development of *BRCA*-associated cancers. However, patients with BC and genetic pathogenic variants do not always have a positive FH, and these criteria miss a large proportion (approximately 50%) of pathogenic variant carriers.^3,4,5^ A genetic testing strategy based on clinical criteria or FH depends on the patient and their physician’s awareness and understanding of the importance of FH, FH accuracy, communication within or between families, and timely referrals to clinical genetics departments. Limited awareness by health care professionals and the public, complexity of the current structure, restricted genetic counseling services, and current testing pathways have fostered restricted access and massive underuse of genetic testing services.^6,7,8^ Only 20% to 30% of eligible patients are referred and access testing, and 97% of estimated carriers in the population remain unidentified,^7^ missing substantial opportunities for precision prevention.^6^ Testing all patients with BC at diagnosis can increase testing access and uptake and identify many more pathogenic variant carriers for screening and prevention. We herein evaluate the cost-effectiveness of this alternative approach of providing genetic testing to all patients with BC regardless of FH.

Knowing a patient’s genetic pathogenic variant status is important for the management and prognosis of BC. After unilateral BC, pathogenic variant carriers can choose contralateral prophylactic mastectomy (CPM) to reduce their risk of developing contralateral BC and opt for surgical prevention of ovarian cancer (OC). Cancer-affected carriers may become eligible for novel drugs (eg, poly [adenosine diphosphate ribose] polymerase [PARP] inhibitors) and other precision medicine–based therapeutics through clinical trials.^9^ A major advantage of genetic testing is enabling testing among relatives of BC pathogenic variant carriers in order to identify unaffected relatives carrying pathogenic variants for early diagnosis and cancer prevention. *BRCA1/BRCA2* carriers have a 17% to 44% risk of developing OC and 69% to 72% risk of BC to 80 years of age.^10^ *PALB2* (OMIM 610355) is a recently established high-penetrance BC gene associated with a 44% BC risk.^11^ A number of risk management options are available for unaffected relatives with pathogenic variants. To reduce OC risk, *BRCA1/BRCA2* pathogenic variant carriers can undergo risk-reducing salpingo-oophorectomy (RRSO).^12,13^ To reduce BC risk, *BRCA1/BRCA2/PALB2* pathogenic variant carriers can be offered enhanced magnetic resonance imaging and mammography screening,^14,15^ risk-reducing mastectomy (RRM),^16^ or chemoprevention with selective estrogen receptor modulators.^17^

Current restricting of testing to FH- or clinical criteria–based selection misses important opportunities to prevent BC and OC in unaffected individuals. In this study, we obtained data from 4 large BC clinical trials and/or research cohorts in the United States, United Kingdom, and Australia. We used modeling to estimate downstream health effects and costs and explore the cost-effectiveness of multigene *BRCA1/BRCA2/PALB2* testing for all cases with BC compared with current *BRCA* testing based on clinical criteria or FH alone. We restrict this analysis to *BRCA1/BRCA2/PALB2*, keeping in mind the principles of the ACCE framework (analytic validity, clinical validity, clinical utility and associated ethical/legal/social implications)^18^ advocated for clinical applicability of genetic testing.^18,19^

## Methods

This analysis received full ethics approval from the Institute of Child Health/Great Ormond Street Hospital Research Ethics Committee as well as the London School of Hygiene and Tropical Medicine Ethics Committee, waiving informed consent for the use of anonymized data. A patient and public involvement statement is found in eMethods 4 in the Supplement.

Data were collected and analyzed from January 1, 2018, through June 8, 2019. We obtained data on FH by age from 11 836 women diagnosed with invasive BC, including (1) 1389 unselected patients with BC older than 45 years who were identified among 57 902 women in the Predicting Risk of Breast Cancer Screening study, a large-scale study within the Greater Manchester UK National Health Service Breast Screening Programme^20^; (2) 2885 patients with BC younger than 40 years from 127 UK hospitals in the Prospective Outcomes in Sporadic vs Hereditary Breast Cancer study^21^; (3) 5892 unselected patients with BC older than 40 years among 132 139 women enrolled in the Kaiser Permanente Washington Breast Cancer Surveillance Consortium registry who underwent mammography screening from 1996 to 2014^22^; and (4) 1670 patients with BC younger and older than 40 years who were randomly selected from the unselected population–based BC cases from the Australian Breast Cancer Family Study.^23^ The proportion of cases fulfilling FH or clinical criteria for testing based on at least a 10% *BRCA1/BRCA2* probability threshold was estimated using standard risk models (eg, BOADICEA [UK and Australian data] and BRCAPRO [US data]).^24,25^ We thus obtained the proportion fulfilling FH or clinical criteria (hereinafter referred to as FH positive) for *BRCA* testing by age group among unselected BC cases in each setting (eTable 1 in the Supplement). The women in these cohorts are predominantly white and representative of a Western population ethnicity (details in eTable 1 in the Supplement). We obtained population-based BC incidence data by age from Cancer Research UK 2015^26^ for the UK analysis and from US Cancer Statistics 2015^27^ for the US analysis. Then we estimated the total number of FH-positive BC cases based on the number of new invasive BC cases by age group in the UK and US populations.

### Model and Genetic Testing Strategy

**Figure 1.**
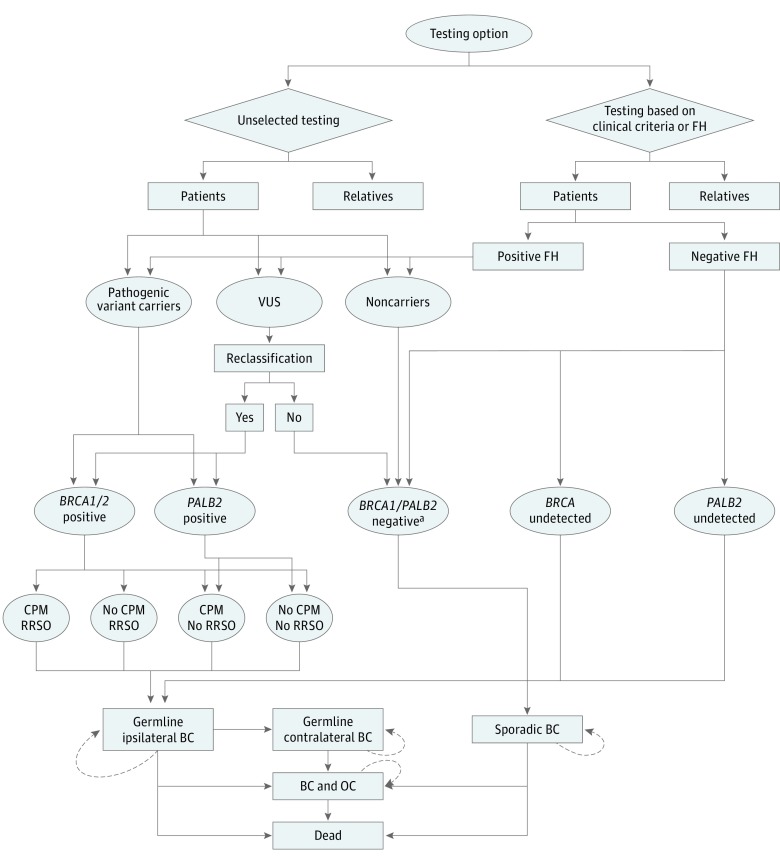
Model Structure Schematic diagram shows the microsimulation model structure for unselected and clinical criteria–or family history (FH)–based panel genetic testing for patients with breast cancer (BC). CPM indicates contralateral prophylactic mastectomy; OC, ovarian cancer; RRSO, risk-reducing salpingo-oophorectomy; and VUS, variant of uncertain significance. ^a^Includes individuals testing negative for *BRCA1/BRCA2/PALB2* mutations and VUS not reclassified as pathologic variants.

**Figure 2.**
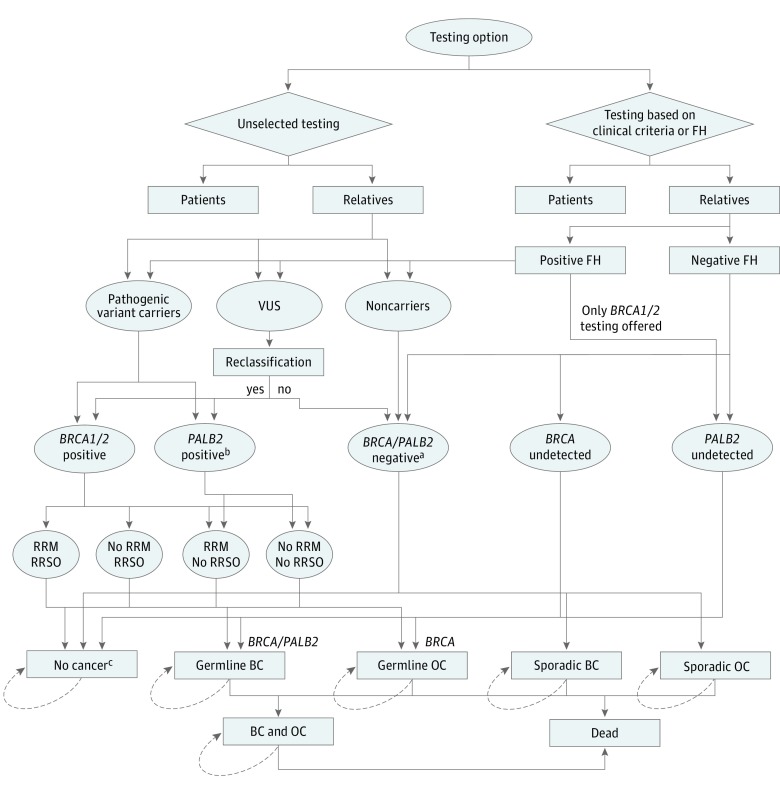
Model Structure Schematic diagram shows the microsimulation model structure for unselected and clinical criteria–or family history (FH)–based panel genetic testing for relatives of patients with breast cancer (BC). CPM indicates contralateral prophylactic mastectomy; OC, ovarian cancer; RRM, risk-reducing mastectomy; RRSO, risk-reducing salpingo-oophorectomy; and VUS, variant of uncertain significance. ^a^Includes individuals testing negative for *BRCA1/BRCA2/PALB2* mutations and VUS not reclassified as pathologic variants. ^b^In the model structure for relatives, *PALB2*-positive individuals are identified only through the unselected testing arm. Relatives in the clinical criteria/FH testing arm only undergo *BRCA1/BRCA2* testing. ^c^Unaffected relatives can progress from no cancer to germline BC (*BRCA1/BRCA2/PALB2*), germline OC (*BRCA1/BRCA2*), sporadic BC, or sporadic OC (or remain in that health state).

We developed an individual-level microsimulation model (illustrated and described in Figure 1 and Figure 2) (TreeAge Pro 2018; TreeAge Software) to analyze costs and effects of *BRCA1/BRCA2/PALB2* testing for all patients with BC (strategy A) compared with the current practice of *BRCA* testing using clinical- or FH-based criteria (≥10% pathogenic variant risk) (strategy B). Microsimulation permits individual heterogeneity in gene types and ages and can track individual patient history if the memory of events (eg, risk-reducing options) affects future cycles. The model assumes all patients in the unselected testing arm (strategy A) and only those fulfilling clinical or FH criteria in strategy B are offered genetic counseling and testing. We assume all eligible patients undergo genetic testing in our base-case analysis. If patients had a *BRCA1/BRCA2/PALB2* pathogenic variant, their first-degree relatives undergo testing for the familial pathogenic variant. If the first-degree relative had a *BRCA1/BRCA2/PALB2* pathogenic variant, second-degree relatives undergo testing. We incorporate a 6.4% variant of uncertain significance (VUS) rate (*BRCA1*, 1.23%; *BRCA2*, 3.29%; and *PALB2*, 1.86%)^28^ and 8.7% pathogenic or likely pathogenic reclassification rate for VUS.^29^

Figure 1 provides a schema of the model with respect to patients with BC. In the unselected testing arm, all patients with BC are offered genetic testing and are classified as pathogenic variant carriers, VUS carriers, or noncarriers. A proportion (8.7%) of patients with VUS results will subsequently get reclassified as pathogenic variant carriers. Identified *BRCA1/BRCA2* pathogenic variant carriers are offered options of CPM and RRSO, and identified *PALB2* pathogenic variant carriers are offered CPM. Depending on the probability of patients undertaking a CPM and/or RRSO, they may progress to germline contralateral BC or both BC and OC. They also have a probability of dying due to germline BC. Patients who do not progress or die would stay in the state of germline ipsilateral BC and undertake the next cycle. Patients with negative findings for *BRCA1/BRCA2/PALB2* have sporadic BC. Age-dependent probabilities allow them to develop sporadic OC and progress to the health state of BC and OC. They also have a probability of dying due to sporadic BC. Women who do not progress to BC and OC or die would stay in the health state of sporadic BC to undertake the next cycle.

In the clinical criteria/FH testing arm, patients with positive FH (fulfilling clinical criteria) undergo genetic testing and are classified as pathogenic variant carriers, VUS carriers, or noncarriers. A proportion of patients with VUS results will subsequently be reclassified as pathogenic variant carriers. Patients with negative FH do not undertake genetic testing. They can be undetected *BRCA1/BRCA2* pathogenic variant carriers, undetected *PALB2* pathogenic variant carriers, or negative for *BRCA1/BRCA2/PALB2*. Options of CPM and/or RRSO and disease progression for identified *BRCA1/BRCA2/PALB2* pathogenic variant carriers and disease progression for patients who are BC negative for *BRCA1/BRCA2/PALB2* is the same as those in the unselected testing arm described above. Undetected *BRCA1/BRCA2* pathogenic variant carriers are not offered CPM or RRSO, and undetected *PALB2* pathogenic variant carriers are not offered CPM. Depending on the baseline risk (no risk-reducing options), they progress to germline contralateral BC or both BC and OC. They also have a probability of dying due to germline BC. Patients who do not progress or die would stay in the state of germline ipsilateral BC and undertake the next cycle.

Figure 2 provides a schema of the model with respect to unaffected relatives identified through cascade testing. Progression through the model depends on the probabilities provided in eTable 2 in the Supplement. In the unselected testing arm, relatives of pathogenic variant carriers with BC are offered *BRCA1/BRCA2/PALB2* genetic testing and classified as pathogenic variant carriers or noncarriers. Relatives of patients with BC and VUS (8.7%) who are reclassified as pathogenic variant carriers are also offered predictive *BRCA1/BRCA2/PALB2* testing. Relatives identified with *BRCA1/BRCA2* pathogenic variants are offered options of RRM and RRSO, and those identified with *PALB2* pathogenic variants are offered RRM. Unaffected relatives can also opt for chemoprevention for BC. Depending on the probability of pathogenic variant carriers undertaking an RRM and/or RRSO (with or without chemoprevention), they progress to germline BC (*BRCA1/BRCA2/PALB2*) or germline OC (*BRCA1/BRCA2*) or stay in the health state of no cancer. They have a probability of background all-cause mortality. Women who are negative for *BRCA1/BRCA2/PALB2* progress to sporadic BC or sporadic OC or stay in the health state of no cancer. They have a probability of background all-cause mortality.

In the clinical criteria/FH testing arm, relatives of identified patients with *BRCA1/BRCA2* mutation undergo predictive *BRCA1/BRCA2* genetic testing. They are classified as pathogenic variant carriers or noncarriers. Relatives of patients with BC and VUS who are reclassified as pathogenic variant carriers also undergo predictive *BRCA1/BRCA2* testing. *PALB2* pathogenic variant carriers cannot be detected when only FH-based *BRCA1/BRCA2* genetic testing is offered. Relatives of patients with negative FH may be undetected *BRCA1/BRCA2* pathogenic variant carriers, undetected *PALB2* pathogenic variant carriers, or negative for *BRCA1/BRCA2/PALB2*. The options of RRM and RRSO for identified carriers are the same as in the unselected testing arm. For identified *BRCA1/BRCA2/PALB2* pathogenic variant carriers and noncarriers (*BRCA1/BRCA2/PALB2* negative), the disease progression is the same as in relatives in the unselected testing arm. Undetected *BRCA1/BRCA2* pathogenic variant carriers are not offered RRM or RRSO, and undetected *PALB2* pathogenic variant carriers are not offered RRM. Depending on the baseline risk, they progress to germline BC or germline OC or stay in a no cancer health state. They also have a probability of background all-cause mortality.

As shown in the model, unaffected *BRCA1/BRCA2/PALB2* pathogenic variant carriers can choose RRM and/or chemoprevention to reduce BC risk and RRSO (*BRCA1/BRCA2* only) to reduce OC risk in addition to undertaking enhanced BC screening. Patients with BC found to have pathogenic variants can opt for CPM. Although initial studies suggested that premenopausal RRSO is associated with reduced BC risk,^13,30,31^ more recent data contradict this observation, especially in *BRCA1*,^32^ raising uncertainty around this issue. We explored no reduction in BC risk in our scenario analysis. We incorporated the excess risk and mortality due to coronary heart disease (CHD) after premenopausal oophorectomy (after RRSO) for premenopausal women who do not take hormone replacement therapy (HRT) (absolute mortality increase, 3.03%).^33,34^ In our model, a hypothetical cohort of patients with BC and their cancer-free relatives can transition to different health states, including no cancer, germline ipsilateral BC, germline contralateral BC, sporadic BC, germline OC, sporadic OC, and both BC and OC. Cancer incidence was estimated by summing the probabilities of pathways ending in OC or BC. The potential population effect was calculated by estimating additional reduction in BC and OC incidence obtained through testing the entire population of BC cases occurring annually in UK and US women. In line with the National Institute of Health and Clinical Excellence (NICE) economic evaluation guidelines, costs and outcomes are discounted at 3.5%.^35^

### Probabilities

Model probabilities for the different pathways are shown in eTable 2 in the Supplement. The age-specific incidences of BC and OC among the general population are obtained from Cancer Research UK 2015^26,36^ and US Cancer statistics 2015.^27^ The age-specific incidence of BC and OC for *BRCA1/BRCA2*^10^ carriers and of BC for *PALB2* carriers,^11^ along with the incidence of contralateral BC after first BC diagnosis,^10^ are obtained from the literature.

### Number and Age Distribution of Relatives

We used the number of new BC cases by age groups in the United Kingdom and United States to calibrate the age distribution of patients in the model.^26,27^ The mean number of first- or second-degree relatives and their ages relative to index cases are derived from data from the Office for National Statistics (in the United Kingdom)^37^ and the National Center for Health Statistics (in the United States)^38^ (details in eTable 3 in the Supplement). We used life tables based on age and sex to estimate the probability of being alive for relatives at different ages and to calculate the number and age distribution of relatives who need to undergo testing.

### Costs

All costs are reported at 2016 prices. The analysis was conducted from payer and societal perspectives. Costs included genetic testing, pretest and posttest genetic counseling,^39,40^ BC, OC, excess CHD, and productivity loss. In line with NICE recommendations, future health care costs not associated with BC, OC, or CHD were not considered.^35^ A summary of costs and detailed explanation are given in eTable 4 in the Supplement (medical costs) and eMethods 1 in the Supplement (costs from productivity loss).

### Life-Years

Our analysis incorporates lifetime risks and long-term consequences to provide a lifetime horizon. Female life tables from the Office of National Statistics (UK women)^41^ and the National Center for Health Statistics (US women)^42^ were used to estimate life expectancy by 80 years for women who did not develop OC or BC. We assumed the median age for undergoing RRM and RRSO in unaffected pathogenic variant carriers was 37 and 40 years, respectively.^43^ We also explored older age at RRM (42 years) and RRSO (46 years) reported in a scenario analysis.^44^ Survival after BC and OC (from diagnosis to death) was modeled using 10-year survival data. Details of survival estimates used are given in eMethods 2 in the Supplement.

### Quality-Adjusted Life-Years

A quality-adjusted life-year (QALY) is a measurement of health outcomes in economic evaluations recommended by NICE. An explanation of QALY and utility scores in the model is given in eMethods 3 in the Supplement.

### Statistical Analysis

In the microsimulation model, we used the number of annual new BC cases (United Kingdom, 54 483; United States, 242 463) and corresponding female relatives (United Kingdom, 215 401; United States, 993 757) by age for running simulations. Internal validation of the model was undertaken through a process of descriptive, technical, and face validity.^45^ We calculated the incremental cost-effectiveness ratio (ICER) by dividing the difference in lifetime costs by the difference in lifetime effects (QALYs) between the 2 strategies as follows: (Cost of Strategy A − Cost of Strategy B)/(Effect of Strategy A − Effect of Strategy B). By comparing the ICER with the willingness-to-pay (WTP) threshold of £30 000/QALY (UK analysis)^46^ and $100 000/QALY (US analysis),^47,48^ we determined whether genetically testing all patients with BC is cost-effective compared with testing based on clinical criteria or FH alone. We undertook a number of scenario analyses, including (1) no reduction in BC risk due to RRSO; (2) nil HRT adherence; (3) lower genetic testing uptake rate (70%) in patients with BC and relatives; (4) 15% *BRCA1/2* pathogenic variant prevalence in patients with BC fulfilling clinical criteria or FH; (5) double cost of genetic counseling (United Kingdom, £40; United States, $80); (6) higher median age for RRM (42 years) and RRSO (46 years) in unaffected pathogenic variant carriers; and (7) the maximum values of cost(s) of genetic testing at which the ICERs reach the WTP thresholds to maintain cost-effectiveness of unselected multigene testing (strategy A).

We performed extensive 1-way and probabilistic sensitivity analyses to explore model parameter uncertainty. In the 1-way sensitivity analysis, each variable or parameter was varied individually to assess the effect on results. Probabilities and utility scores were varied by their 95% CIs or range where available or by ±10%, and costs were varied by ±30%. In the probabilistic sensitivity analysis, all of the input variables were varied simultaneously (as recommended by NICE).^49^ As suggested in the literature,^50^ costs were given a γ distribution; quality of life, a log-normal distribution; and probability, a β distribution. For probabilistic sensitivity analysis, we obtained 1000 estimates of incremental costs and effects by sampling from the distributions of each variable. A cost-effectiveness acceptability curve was then plotted to show the probability of genetically testing all patients, with BC (strategy A) being cost-effective at different WTP thresholds.

## Results

Compared with the current practice of genetic testing based on clinical criteria or FH, offering unselected multigene testing for all patients diagnosed annually with BC (54 483 in the United Kingdom and 242 463 in the United States) and subsequent predictive/cascade testing of relatives (strategy A) was highly cost-effective. The ICER for the UK payer perspective was £10 464/QALY (credible interval, £8347/QALY to £28 965/QALY) and for the societal perspective, £7216/QALY (credible interval, £6194/QALY to £23 575/QALY). The ICER for the US payer perspective was $65 661 per QALY (credible interval, $46 613/QALY to $248 185/QALY) and for the societal perspective, $61 618/QALY (credible interval, $42 927/QALY to $221 781/QALY). The lifetime costs, QALYs, and population effects (reduced cancer incidence and deaths) for UK and US women are shown in Table 1 and Table 2. Strategy A was associated with an additional 419-day increase in life expectancy for UK and 298 days for US *BRCA1/BRCA2/PALB2* pathogenic variant carriers. One year’s unselected genetic testing of all patients with BC could prevent an additional 1142 BC cases and 959 OC cases in the United Kingdom and 5478 BC cases and 4255 OC cases in the United States (Table 2). This finding corresponds to averting 633 deaths due to cancer in UK populations and 2406 deaths due to cancer in US populations during a lifetime horizon (Table 2). The corresponding excess deaths due to heart disease were 8 in UK and 35 in US women annually.

**Table 1.** Lifetime Discounted Costs and Effects per Woman and ICER After Genetic Testing for All Patients With BC^a^

Country	Testing All Patients With BC				Testing Based on Family History				ICER			
	Health Effects		Costs^b^		Health Effects		Costs^b^		Cost/LYG^b^		Cost/QALY^b^	
	LYGs	QALYs	Payer	Societal	LYGs	QALYs	Payer	Societal	Payer	Societal	Payer	Societal
**Baseline**												
United Kingdom	18.772	17.941	7213	11 147	18.755	17.922	7016	11 011	11 817	8149	10 464	7216
United States	18.652	17.813	32 721	36 561	18.639	17.798	31 724	35 625	82 789	77 691	65 661	61 618
**No Reduction in BC Risk Due to RRSO**^c^												
United Kingdom	18.772	17.941	7214	11 148	18.755	17.922	7016	11 011	11 846	8201	10 532	7291
United States	18.652	17.813	32 724	36 564	18.639	17.798	31 724	35 625	82 902	77 844	66 136	62 102
**No HRT Adherence**^d^												
United Kingdom	18.771	17.940	7218	11 152	18.755	17.922	7016	11 011	12 706	8846	11 303	7870
United States	18.651	17.812	33 013	36 852	18.639	17.798	31 751	35 652	113 342	107 823	89 705	85 337
**Lower Uptake Rate of Genetic Testing in Patients and Relatives**^e^												
United Kingdom	18.766	17.934	7132	11 096	18.755	17.922	7009	11 007	11 363	8319	10 991	8046
United States	18.644	17.804	32 299	36 170	18.637	17.796	31 691	35 595	80 043	75 849	71 006	67 285
**15% Probability of Being a *BRCA* Carrier in Patients With Positive FH**^f^												
United Kingdom	18.771	17.941	7213	11 147	18.755	17.923	7022	11 015	11 973	8293	10 585	7332
United States	18.653	17.814	32 723	36 563	18.641	17.800	31 759	35 657	84 453	79 326	66 694	62 646
**Double Cost of Counseling**^g^												
United Kingdom	18.772	17.941	7220	11 154	18.755	17.922	7016	11 011	12 189	8521	10 794	7546
United States	18.652	17.813	32 734	36 574	18.639	17.798	31 725	35 625	83 798	78 701	66 462	62 419
**Older Ages for RRM and RRSO in Unaffected Pathogenic Variant Carriers**^h^												
United Kingdom	18.770	17.938	7216	11 165	18.755	17.922	7016	11 013	13 181	10 043	12 214	9306
United States	18.650	17.811	32 722	36 578	18.639	17.798	31 720	35 622	92 304	88 063	77 715	74 144

Abbreviations: BC, breast cancer; FH, family history; HRT, hormone replacement therapy; ICER, incremental cost-effectiveness ratio; LYG, life-years gained; QALY, quality-adjusted life-year; RRM, risk-reducing mastectomy; RRSO, risk-reducing salpingo-oophorectomy.

^a^ Costs and outcomes are discounted at 3.5%. Data are given at baseline (for the base case) and for separate scenarios.

^b^ Costs are given in dollars for the United States and pounds sterling for the United Kingdom.

^c^ Probability P 15 = 1 (eTable 2 in the Supplement).

^d^ Probability P 21 = 0 (eTable 2 in the Supplement).

^e^ Indicates a genetic testing uptake rate of 70%.

^f^ Probability P 4 = 0.15 (eTable 2 in the Supplement).

^g^ Indicates £40 in the United Kingdom and $80 in the United States.

^h^ Indicates ages 42 and 46 years for RRM and RRSO, respectively.

**Table 2.** Population Effect of Genetic Testing for Patients With BC

Estimated Effect	Testing in All Patients With BC		Testing Based on FH		Differences		
	Patients	Relatives	Patients	Relatives	Patients	Relatives	Total
UK germline cancer							
No. of BC cases	364^a^	1965	684^a^	2787	320^a^	822	1142
No. of OC cases	447	1882	871	2417	424	535	959
No. of BC and OC deaths	451	988	748	1325	296	337	633
US germline cancer							
No. of BC cases	1639^a^	8727	3230^a^	12 614	1591^a^	3887	5478
No. of OC cases	2087	8655	3916	11 081	1829	2426	4255
No. of BC and OC deaths	1555	4168	2621	5508	1066	1340	2406

Abbreviations: BC, breast cancer; FH, family history; OC, ovarian cancer.

^a^ Indicates contralateral BC cases in patients with unilateral BC.

**Figure 3.**
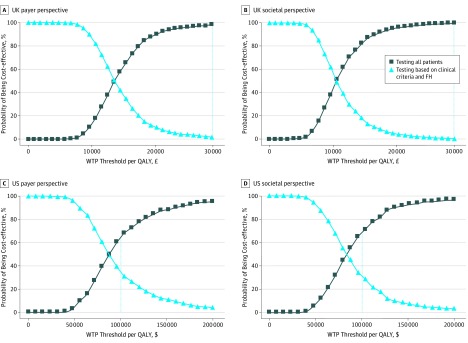
Cost-effectiveness Acceptability Curves (Probabilistic Sensitivity Analyses) Probabilistic sensitivity analysis in which all model parameters/variables are varied simultaneously across their distributions to further explore model uncertainty. The results of 1000 simulations were plotted on a cost-effectiveness acceptability curve showing the proportion of simulations that indicated that the intervention was cost-effective at different willingness-to-pay (WTP) thresholds. A and B, The dotted line marks the proportion of simulations found to be cost-effective at the WTP threshold of £30 000 per quality-adjusted life-year (QALY) in the UK analysis. At the £30 000/QALY WTP threshold from the payer perspective, 2% simulations are cost-effective for testing based on clinical criteria or family history (FH) and 98% simulations are cost-effective for unselected genetic testing; from the societal perspective, 1% simulations are cost-effective for testing based on clinical criteria or FH and 99% simulations are cost-effective for unselected genetic testing. C and D, The dotted line marks the proportion of simulations found to be cost-effective at the WTP threshold of $100 000/QALY in the US analysis. At the $100 000/QALY WTP threshold from the payer perspective, 36% simulations are cost-effective for testing based on clinical criteria or FH and 64% simulations are cost-effective for unselected genetic testing; from the societal perspective, 32% simulations are cost-effective for testing based on clinical criteria or FH and 68% simulations are cost-effective for unselected genetic testing.

The 1-way sensitivity analysis (eFigure 1A-D in the Supplement) indicates that pathogenic variant prevalence, costs, utility scores, and transition probabilities had little individual influence on the cost-effectiveness of unselected genetic testing (strategy A) from a payer or a societal perspective. Scatterplots for the UK and US analyses are given in eFigure 2 in the Supplement and show that all simulations and iterations lie in the northeast quadrant, indicating unselected testing was always more effective. The ICERs are lower than the UK and US WTP thresholds at the upper and lower limits of these variables. Probabilistic sensitivity analysis (Figure 3) shows that at the £30 000/QALY or $100 000/QALY thresholds, 98% (UK payer perspective), 99% (UK societal perspective), 64% (US payer perspective), or 68% (US societal perspective) of simulations indicate that unselected genetic testing is cost-effective compared with testing based on FH or clinical criteria.

The number of pathogenic variant carriers among unaffected female relatives identified through cascade testing was 1.41 in the United Kingdom and 1.46 in the United States per index pathogenic variant carrier with BC (details in eTable 4 in the Supplement). Scenario analyses are presented in Table 1. Unselected testing was cost-effective from payer and societal perspectives, even with alternative scenarios of no reduction in BC risk due to RRSO (ICER payer perspective, £10 532/QALY or $66 136/QALY; ICER societal perspective, £7291/QALY or $62 102/QALY); nil HRT adherence (ICER payer perspective, £11 303/QALY or $89 705/QALY; ICER societal perspective, £7870/QALY or $85 337/QALY); and lower (70%) genetic testing uptake rate in patients with BC and relatives (ICER payer perspective, £10 991/QALY or $71 006/QALY; ICER societal perspective, £8046/QALY or $67 285/QALY). Although the probability of being a *BRCA1/BRCA2* carrier in those fulfilling FH or clinical genetic testing criteria was reported at approximately 10%,^51,52^ we also explored a scenario of overall 15% *BRCA1/BRCA2* carrier probability. This variable had only a minimal effect on ICERs from the payer (£10 585/QALY) and societal (£7332/QALY) perspectives among UK women and from the payer ($66 694/QALY) and societal ($62 646/QALY) perspectives among US women. The upper limit of genetic testing costs at which unselected genetic testing for all patients with BC would still remain cost-effective at the established WTP thresholds was approximately £1626 from the payer perspective and £1868 from the societal perspective for the UK health system and $2432 from the payer perspective and $2679 from the societal perspective for the US health system.

Lower RRSO and RRM rates are reported in some populations.^53^ The minimum RRSO uptake rate to maintain cost-effectiveness was 29% from the payer perspective or 28% from the societal perspective for the United States (ICER, $100 000/QALY), but unselected BC genetic testing was cost-effective in the United Kingdom even if the RRSO rate was nil (ICER from the payer perspective, £26 392/QALY; ICER from the societal perspective, £23 802/QALY). The strategy was cost-effective even if RRM rates in unaffected relatives approached 0 (UK ICER from the payer perspective, £9969/QALY; UK ICER from the societal perspective, £7041/QALY; US ICER from the payer perspective, $67 235/QALY; US ICER from the societal perspective, $63 643/QALY). However, if RRM uptake was 0, then the minimum RRSO uptake rate to maintain cost-effectiveness at the WTP thresholds (United States, $100 000/QALY; United Kingdom, £30 000/QALY) was 33% (payer perspective) or 32% (societal perspective) in the US health system and 5% (payer perspective) or 4% (societal perspective) in the UK health system.

## Discussion

Our analysis addresses a topical and important issue of unselected multigene testing for all patients with BC. We show for the first time, to our knowledge, that multigene testing for high-penetrance BC pathogenic variants of well-established clinical utility is more cost-effective and outperforms standard *BRCA* testing driven by clinical criteria or FH alone. Moving toward such a program could lead to 1142 fewer BC cases, 959 fewer OC cases, and 663 fewer deaths due to BC or OC in UK women and 5478 fewer BC cases, 4255 fewer OC cases, and 2406 fewer deaths due to BC or OC in US women annually. Our study provides QALY-based health outcomes that justify the cost differences between the 2 strategies that are needed for health care professionals, providers, and policy makers to guide or direct resource allocation. The ICERs (£10 464/QALY and £7216/QALY in the United Kingdom and $65 661/QALY and $61 618/QALY in the United States) lie well below the established cost-effectiveness thresholds for the UK (£20 000/QALY to £30 000/QALY) and the US ($100 000/QALY) health systems. Continuing with the current FH- or clinical criteria–based policy reflects important opportunities missed for BC and OC prevention.

### Comparison With Other Studies

Although earlier studies have reported cost-effectiveness of *BRCA* testing at the 10% pretest probability threshold,^54^ we report cost-effectiveness of unselected *BRCA/PALB2* testing irrespective of a priori mutation probability. Our findings are in line with a recent, small Norwegian study (535 patients) showing cost-effectiveness of *BRCA* testing for all patients with BC.^5^ Our study is broader in scope and draws on a much larger sample size of population-based UK, US, and Australian patients with BC. Testing at cancer diagnosis has now moved toward multigene testing. *PALB2* is associated with nonsyndromic, quasi-mendelian BC susceptibility (BC risk, 44%), and magnetic resonance imaging screening and RRM are now offered for pathogenic variants. Other high-risk genes are identifiable as pleiotropic syndromic (*STK11*, *PTEN, or p53*) or associated with only a small subset (lobular), and all are very rare.^19^ In addition, reliable risk estimates corrected for ascertainment bias are lacking.^19^ Although *ATM* and *CHEK2* are included in some commercial panels, clinical testing for these genes is not routine in most centers. Risks conferred by these pathogenic variants are lower (relative risk, approximately 1.5-2.0), and although National Comprehensive Cancer Network guidelines support breast screening, RRM is not routinely offered, FH needs incorporation into risk assessment and management, and many health care professionals believe that they fall below the clinical intervention threshold.^19^ Hence, we incorporated *PALB2* along with *BRCA* but excluded other genes.

### Implications

The current health care model of testing based on clinical criteria or FH has numerous limitations. It misses a large proportion of pathogenic variant carriers who fall below the current clinical threshold.^3,5^ The current system is plagued by massive underuse of genetic testing and missed opportunities for BC and OC screening and prevention.^6,7^ Moving toward unselected BC testing may give an impetus for prevention in unaffected family members along with clinical implications for the patient with BC. Pathogenic variant carriers with newly diagnosed BC can opt for bilateral mastectomy rather than breast conservation at initial BC surgery. Bilateral mastectomy reduces contralateral BC risk, may provide better options for breast reconstruction, and may obviate the need for adjuvant radiotherapy.^55^ The patients also become eligible for therapeutic options, such as PARP inhibitors. Addressing the increasing burden of long-term and chronic disease, including cancer, is one of the world’s greatest public health challenges and is important for future viability of health systems across the world.^56^ The Milken Institute estimates that improving prevention can cut millions of cases of chronic disease and reduce treatment costs by billions.^57^ The applicability of genomics to medicine is growing and expanding. Moving toward unselected multigene testing for patients with BC can provide a huge stimulus for precision prevention.

Existing genetic counseling services operating through high-risk cancer genetics clinics do not have the resources or manpower to deliver unselected genetic testing for all patients with BC given the large numbers of patients who receive a diagnosis annually. Hence, newer context-specific delivery models will be needed for implementing this approach. These models may require pretest counseling to be undertaken by nongenetic health care professionals who will need to be trained for this. This approach of mainstreaming genetic counseling and testing has recently been successfully implemented in OC treatment pathways.^58,59^ Oncologists, surgeons, and clinical nurse specialists have provided pretest counseling and genetic testing,^58,59^ with genetic services focusing on posttest counseling and support for women carrying pathogenic variants. A similar approach could work for patients with BC. Examples of other delivery options include a genetics service–coordinated nurse-led model,^60^ a genetics-embedded model (genetics health care professional or counselor embedded in the cancer clinic),^61,62^ and telephone counseling^40,63,64^ or telegenetics services^65^ for genetic counseling and testing.

Going forward, most health care professionals who practice medicine will need an increased understanding of genetics and ability to counsel patients about this topic.^8,66^ As the volume of testing rises, the number of mutations and VUS being diagnosed along with the need for correct interpretation and management will increase. Implementation will need to be accompanied by a process of training and education for relevant physicians and other health care professionals involved in the care pathway so that they can understand the implications for management, including that of VUS. This process is critical to ensure best evidence–based care^67^ and to avoid unintended or inappropriate management, such as downstream predictive testing, screening, or prevention in VUS cases.^68^ Updated guidelines need to reflect the importance of appropriate management. Appropriate clinical decision support tools can facilitate this transformation. Another potential bottleneck to address is laboratory infrastructure to manage increased sample throughput. Although some health systems have adequate capacity, others may lack this infrastructure. Future research needs to evaluate the effects and downstream outcomes of various context-specific genetic testing implementation and management pathways for patients with BC.

### Strengths and Limitations

Our study has several strengths. The model incorporates unselected BC data from large population-based studies, up-to-date information from the Genetics Cancer Prediction Through Population Screening study,^69^ published literature, and public databases such as those of the Office for National Statistics (United Kingdom),^37,41^ National Center for Health Statistics (United States),^38,42^ and Cancer Research UK.^26,36^ We use the current standard of clinical care (approach based on clinical criteria or FH) as the comparator and present analyses from the payer and societal perspectives. Our analysis follows NICE recommendations: QALYs to measure health outcomes; cost-effectiveness analysis for health economic evaluation,^49^ integration of utility scores, discounting costs and outcomes (rate, 3.5%), sufficiently long horizon (lifetime) to uncover important differences in costs and outcomes, and extensive and thorough 1-way and probabilistic sensitivity analyses that support robustness and accuracy of results (eFigure 1 in the Supplement and Figure 2). We include a detriment for CHD mortality.^33^ Our costs include genetic testing, VUS management, pretest and posttest genetic counseling, HRT use, and protection from osteoporosis.

Our study has limitations related to modeling assumptions. Our baseline model assumes that all women with BC and their unaffected relatives undergo genetic testing. Although very high (≤98%), genetic testing rates are reported in unselected genetic testing at OC diagnosis, and corresponding genetic testing uptake data in unselected patients with BC are not well established. Our scenario analysis reconfirms cost-effectiveness at lower (70%) uptake rates. Although our base model incorporates reduction in BC risk with premenopausal oophorectomy in keeping with many initial analyses,^13,30,31,70^ recent uncertainty surrounds this.^32^ Our scenario analysis reconfirms cost-effectiveness even without this benefit. Although genetic testing costs have fallen drastically, some health care providers charge higher prices than our base-case assumption. Nevertheless, unselected BC testing would remain cost-effective even at £1626 to £1868 in the United Kingdom or $2432 to $2679 in the United States, which is many times greater than costs charged by most health care providers today. Another limitation is that our model incorporates data predominantly from white women, which can limit interpretation of generalizability to nonwhite populations.

Although we have incorporated disutility for RRSO and RRM, surgical prevention might have associated complications (RRSO, approximately 3%-4%^71^; RRM, approximately 21%)^72,73^ that need to be factored into the informed consent and decision-making process. Although premenopausal RRSO is not associated with worsening general quality of life, poorer sexual function is reported (despite HRT).^74,75^ This outcome is compensated by extremely high satisfaction rates and reduction in perceived cancer risk and/or worry with RRSO.^74,76^ Risk-reducing mastectomy is negatively associated with sexual pleasure and body image. These disadvantages may be offset by reduced anxiety, improved social activity,^77^ good cosmetic satisfaction rates,^78,79^ and lack of negative impact on sexual activity/habit/discomfort,^77^ anxiety/depression, or generic quality of life.^77,80,81^ We confirmed that unselected multigene testing remains cost-effective at recently reported older ages of RRM and RRSO.^44^ The surgical prevention (RRM and RRSO) rates used are based on established UK and US data.^43,82^ However, these rates can vary, with lower rates reported in some populations.^53^ Those ascertained from population testing may have lower BC risks and result in lower uptake, particularly in the absence of death due to BC and heavy cancer burden in the family. Our scenario analyses show that unselected testing remains cost-effective at lower RRSO and RRM rates.

## Conclusions

This study’s findings suggest that unselected multigene testing for BC susceptibility genes *BRCA1/BRCA2/PALB2* can substantially reduce future BC and OC cases and related deaths compared with the current clinical strategy. Our analysis suggests that an unselected testing strategy is extremely cost-effective for UK and US health systems and provides a basis for change in current guidelines and policy to implement this strategy.

## Notes

Supplement. **eTable 1.** Family History Information in Breast Cancer Cases **eTable 2.** Probabilities of Different Pathways in the Model and Explanations **eTable 3.** Generating Cohort of Relatives **eTable 4.** Summary of Medical Costs Used in the Model (2016 Prices) and Explanation **eMethods 1.** Examination of Productivity Loss **eMethods 2.** Estimates for Age of Onset and Survival for Breast and Ovarian Cancers **eMethods 3.** Quality-Adjusted Life-Years (QALYs) and Utility Scores **eMethods 4.** Patient and Public Involvement Statement **eFigure 1.** Tornado Diagram of 1-Way Sensitivity Analysis **eFigure 2.** Scatterplots for Incremental Discounted Lifetime Costs and Effects of Unselected Multigene Testing Compared With BRCA Testing Based on Family History and Clinical Criteria **eReferences.**
