# Supplementary material for: A Cost-effectiveness Analysis of Multigene Testing for All Patients With Breast Cancer
Source: JAMA Oncol. 2019 Oct 3;5(12):1718–30. doi: 10.1001/jamaoncol.2019.3323 (PMC6777250; doi:10.1001/jamaoncol.2019.3323)
Supplement: Supplement. — eTable 1. Family History Information in Breast Cancer Cases eTable 2. Probabilities of Different Pathways in the Model and Explanations eTable 3. Generating Cohort of Relatives eTable 4. Summary of Medical Costs Used in the Model (2016 Prices) and Explanation eMethods 1. Examination of Productivity Loss eMethods 2. Estimates for Age of Onset and Survival for Breast and Ovarian Cancers eMethods 3. Quality-Adjusted Life-Years (QALYs) and Utility Scores eMethods 4. Patient and Public Involvement Statement eFigure 1. Tornado Diagram of 1-Way Sensitivity Analysis eFigure 2. Scatterplots for Incremental Discounted Lifetime Costs and Effects of Unselected Multigene Testing Compared With BRCA Testing Based on Family History and Clinical Criteria eReferences. [file jamaoncol-5-1718-s001.pdf]

Supplementary Online Content

Sun L, Brentnall A, Patel S, et al. A cost-effectiveness analysis of multigene testing for all patients with breast cancer. *JAMA Oncol.* Published online October 3, 2019. doi:10.1001/jamaoncol.2019.3323

eTable 1. Family History Information in Breast Cancer Cases
eTable 2. Probabilities of Different Pathways in the Model and Explanations
eTable 3. Generating Cohort of Relatives
eTable 4. Summary of Medical Costs Used in the Model (2016 Prices) and Explanation
eMethods 1. Examination of Productivity Loss
eMethods 2. Estimates for Age of Onset and Survival for Breast and Ovarian Cancers
eMethods 3. Quality-Adjusted Life-Years (QALYs) and Utility Scores
eMethods 4. Patient and Public Involvement Statement
eFigure 1. Tornado Diagram of 1-Way Sensitivity Analysis
eFigure 2. Scatterplots for Incremental Discounted Lifetime Costs and Effects of Unselected Multigene Testing Compared With BRCA Testing Based on Family History and Clinical Criteria
eReferences.

This supplementary material has been provided by the authors to give readers additional information about their work.

© 2019 American Medical Association. All rights reserved.

eTable 1. Family History Information in Breast Cancer Cases

	POSH		BCSC		PROCAS		ABCFS		Overall			BC cases in population		FH+ BC cases in population	
Age	Total BC cases	FH+ BC cases	Total BC cases	FH+ BC cases	Total BC cases	FH+ BC cases	Total BC cases	FH+ BC cases	Total BC cases	FH+ BC cases	Proportion of FH+ in BC cases	UK	USA	UK	USA
<40	2885	548	0	0	0	0	890	44	3775	592	15.7%	2194	10679	344	1675
40-44	0	0	262	40	0	0	198	5	460	45	9.8%	2754	12818	269	1254
45-49	0	0	578	58	59	3	202	7	839	68	8.1%	5228	19949	424	1617
50-54	0	0	874	71	289	10	191	2	1354	83	6.1%	6391	25506	392	1564
55-59	0	0	978	49	241	9	156	0	1375	58	4.2%	5410	29388	228	1240
60-64	0	0	1036	42	290	3	17	0	1343	45	3.4%	6276	33231	210	1113
65-70	0	0	909	33	323	5	16	0	1248	38	3.0%	7672	35651	234	1086
70-74	0	0	698	22	156	5	0	0	854	27	3.2%	5127	28571	162	903
75+	0	0	557	17	31	2	0	0	588	19	3.2%	13431	46670	434	1508
Total	2885	548	5892	332	1389	37	1670	58	11836	975	8.2%	54483	242463	2697	11959

ABCFS - Australian Breast Cancer Family Study, BC – breast cancer, FH+ – family history positive, POSH – Prospective Outcomes in Sporadic versus Hereditary breast cancer study, PROCAS - Predicting Risk of Breast Cancer Screening study

The numbers of breast cancer cases by age group in the population are obtained from Cancer Research UK 2015[1] and US Cancer Statistics 2015[2]. The probability of having a positive FH among unselected patients is 2697/54483 in the UK and 11959/242463 in the USA.

<u>Ethnicity Distribution:</u>

PROCAS study: 91% White, 1.54% Asian, 1.16% Black, 0.9% Jewish, 0.5% Mixed, 1.68% other and 3.23% Unknown.[3]

POSH study: 92.7% White, 3.7% Black, 3% Asian, 0.7% from ‘other’ ethnic groups, and 0.5% Mixed.[4]

BSCS registry: 85% White, 7% Asian, 3% Black, 3% Mixed, 1% Native American Indian/Alaskan, 1% Other and <1% Unknown.[5]

ABCFS: 92% White, 5.9% Asian, 1% Maori/Aboriginal/Pacific, 1.3% Other

© 2019 American Medical Association. All rights reserved.

eTable 2. Probabilities of Different Pathways in the Model and Explanations

Probability	Value	(95% CI) [Range]	Description	Source
P1	0.0464	(0.044,0.049)	*BRCA1/BRCA2* mutation prevalence in unselected breast cancer patients	6
P2	0.0089	(0.008,0.010)	*PALB2* mutation prevalence in unselected breast cancer patients	6
P3	0.0495	(0.048,0.051)	Probability of having a positive FH among unselected patients	7-9
P4	0.1	--	*BRCA1/BRCA2* mutation prevalence in FH-positive patients	10
P5	0.008	(0.005,0.013)	*PALB2* mutation prevalence in FH-positive patients	11
P6	0.0453	(0.0350, 0.0585)	*BRCA1/BRCA2* VUS prevalence in breast cancer patients	12
P7	0.0186	(0.0130, 0.0264)	*PALB2* VUS prevalence in breast cancer patients	12
P8	0.0869	(0.0755, 0.0999)	Reclassification rate of VUS	13
P9	0.47	(0.34,0.56)	Uptake of RRM in unaffected mutation carriers	14
P10	0.539	(0.442,0.636)	Uptake of CPM in carriers with breast cancer	15
P11	0.55	(0.45,0.64)	Uptake of RRSO in unaffected carriers	16
P12	0.567	(0.506,0.629)	Uptake of RRSO in carriers with breast cancer	17
P13	0.911	(0.62,0.98)	Reduction in breast cancer risk from RRM without RRSO in unaffected mutation carriers	18
P14	0.95	(0.78,0.99)	Reduction in breast cancer risk from RRM with RRSO in unaffected mutation carriers	18
P15	0.49	(0.37,0.65)	HR for breast cancer from RRSO alone in unaffected mutation carriers	19
P16	0.18	(0.07,0.45)	HR for contralateral breast cancer risk from CPM after breast cancer diagnosis	15
P17	0.35	(0.20,0.61)	HR for contralateral breast cancer risk from RRSO after breast cancer diagnosis	20
P18	0.96	[0.8,0.96]	Reduction in ovarian cancer risk from RRSO	19,21
P19	0.46	(0.27,0.79)	HR for breast cancer survival from RRSO	22
P20	0.37	(0.17,0.80)	HR for breast cancer survival from CPM	15
P21	0.8	(0.76,0.83)	Compliance of HRT	23
P22	0.71	(0.60,0.83)	HR of breast cancer risk from chemoprevention	24
P23	0.163	(0.136,0.19)	Uptake of breast cancer chemoprevention	25
P24	0.0072	(0.0068,0.0076)	Annual excess risk of developing CHD after RRSO	26
P25	0.0303	(0.011,0.043)	Cumulative mortality from CHD after RRSO without HRT	26

95%CI - 95% confidence interval, CHD - coronary heart disease, CPM – contralateral prophylactic mastectomy, FH - family history, HR - Hazard Ratio, HRT - hormone replacement therapy, RRSO – risk-reducing salpingo-oophorectomy, RRM – risk-reducing mastectomy, VUS – variant of uncertain significance.

© 2019 American Medical Association. All rights reserved.

Explanations:

P1-P2: The probabilities of carrying a *BRCA1/BRCA2/PALB2* mutation in unselected breast cancer patients are taken from a US analysis by Buys et al 2017 among 35,409 women with a single diagnosis of breast cancer undergoing clinical genetic testing [6].

P3: We obtained the proportion of having a positive family history (having ≥10% *BRCA1/BRCA2* mutation risk) among unselected breast cancer cases from Kaiser Permanente Washington breast imaging registry [7], POSH study [8], and PROCAS study [9] and unselected population based breast cancer cases from the ABFCS.[27] Then we used the number of breast cancer cases by age from Cancer Research UK[1] to calculate the overall proportion of having a positive family history among unselected breast cancer patients. Correspondingly the breast cancer cases by age for the US was obtained from the United States Cancer Statistics [2].

P4: The overall *BRCA1/BRCA2* mutation prevalence (10%) among FH positive breast cancer patients is based on the current testing guideline.

P5: The probability of carrying a *PALB2* mutation in breast cancer patients with a positive FH is taken from Slavin 2017.[11] The *BRCA1/BRCA2* mutation probability in FH positive individuals is 0.1, which is the threshold for genetic testing in the current guideline. Among *BRCA1/BRCA2* negative familial breast cancer patients (90% of patients), the *PALB2* prevalence is 0.89%. Therefore, the overall PALB2 prevalence in all FH positive breast cancer patients is 0.8% or 0.008.

P6-P7: We obtained the *BRCA1/BRA2/PALB2* VUS prevalence from a systematic review and meta-analysis by van Marcke et al 2018 including 1,870 breast cancer patients[12]. VUS rate to be 1.23% for *BRCA1*, 3.29% for *BRCA2* and 1.86% for *PALB2* in high-risk breast cancer patients.[12] This gives a total VUS rate of 6.4%.[12]

P8: The reclassification rate of VUS is taken from Mersch et al 2018[13]. 8.69% of VUS (178 of 2048) were upgraded to pathogenic or likely pathogenic variants.

P9: The probability that unaffected carriers will undergo RRM is taken from an analysis of UK *BRCA1/2* carriers by Evans et al 2009 [14]. A composite uptake rate for *BRCA1* (60% RRM rate) and *BRCA2* (43% RRM rate) carriers weighted for the relative prevalence of *BRCA1 and BRCA2* mutations was computed [14].

© 2019 American Medical Association. All rights reserved.

P10: The uptake of CPM in *BRCA1/BRCA2* women diagnosed with unilateral breast cancer is obtained from a cohort study by Evans et al 2013 in the UK [15].

P11: The uptake of RRSO in unaffected *BRCA1/BRCA2* carriers is taken from a study among high-risk UK women [16].

P12: The uptake of RRSO in women with *BRCA1/BRCA2* breast cancer is taken from Kauff et al 2008[17].

P13: The reduction in breast cancer risk from RRM in *BRCA1/BRCA2* mutation carriers not undergoing RRSO is taken from the PROSE study data by Rebbeck et al 2004 [18].

P14: The reduction in breast cancer risk in *BRCA1/BRCA2* mutation carriers undergoing RRM and RRSO is taken from the PROSE study data by Rebbeck et al 2004 [18].

P15: The Hazard Ratio for breast cancer in pre-menopausal unaffected *BRCA1/BRCA2* women undergoing RRSO alone is taken from a meta-analysis by Rebbeck et al 2009 [19].

P16: The Hazard Ratio for contralateral breast cancer risk from CPM in women with *BRCA1/BRCA2*-associated breast cancer is obtained from Evans 2013 [15].

P17: The Hazard Ratio for contralateral breast cancer risk from RRSO in *BRCA1/BRCA2* mutation carriers after breast cancer diagnosis is obtained from a UK study by Basu 2015 [20], using data from the regional genetics service and the family history clinic at the Genesis Breast Cancer Prevention Centre in Manchester.

P18: The reduction in ovarian cancer risk obtained from RRSO is taken from previous studies which report a 4% residual-risk of primary peritoneal cancer following RRSO [21].

P19: The Hazard Ratio for breast cancer survival from RRSO is obtained from Metcalfe 2015 [22].

P20: The Hazard Ratio for breast cancer survival from CPM is obtained from Evans 2013 [15].

P21: HRT compliance rate is obtained from a UK cohort (Read et al, 2010) [23].

P22: The Hazard Ratio for breast cancer risk from chemoprevention in high-risk women is obtained from the extended long-term follow-up of the IBIS-I breast cancer prevention trial (Cuzick et al 2015) [24].

P23: The uptake of breast cancer chemoprevention is obtained from a recent meta-analysis by Smith et al 2016 [25].

© 2019 American Medical Association. All rights reserved.

P24: Excess risk of CHD after RRSO is estimated using data from Parker 2013 [26]. The absolute excess CHD incidence is obtained by subtracting CHD incidence in women undergoing RRSO from those not.

P25: The risk of CHD mortality is obtained from the Nurses Health Study (Parker et al 2013) [26]. Death from CHD is reported in 1 in 33 pre-menopausal women undergoing RRSO and not taking HRT [26].

© 2019 American Medical Association. All rights reserved.

eTable 3. Generating Cohort of Relatives

**Country**	**UK**				**USA**			
**First-degree relatives**	Mother	Father	Siblings	Children	Mother	Father	Siblings	Children
Average number	1	1	0.91	1.91	1	1	0.99	1.99
Age relative to index case	30	32	0	-30	29	31	0	-29
Sex, probability female	100%	0%	50.78%	50.78%	100%	0%	50.76%	50.76%
Probability mutation	50%	50%	50%	50%	50%	50%	50%	50%
**Second-degree relatives**	Grandparents	Uncle/aunts	Nieces/nephews	Grandchildren	Grandparents	Uncle/aunts	Nieces/nephews	Grandchildren
Average number	4	1.82	1.74	3.65	4	1.98	1.97	3.96
Age relative to first-degree relatives	30	0	-30	-30	29	0	-29	-29
Sex, probability female	50%	50.76%	50.76%	50.76%	50%	50.76%	50.76%	50.76%
Probability mutation	25%	25%	25%	25%	25%	25%	25%	25%
Reference	Office for National Statistics [28]				National Centre for Health Statistics [29]			

The average number of first or second-degree relatives, ages relative to index cases, and the probability of being female are derived from data from the Office for National Statistics (UK) [28] and the National Centre for Health Statistics (USA).[29] The number of breast cancer cases by age group is reported by Cancer

© 2019 American Medical Association. All rights reserved.

Research UK 2015[1] and US Cancer Statistics 2015[2]. Based on the average number of relatives and the age relative to the index cases (see table above), we calculated the number of first-/second-degree relatives at different ages. Then we used the lifetables based on age and gender [30,31] to obtain the probability of being alive for relatives at different ages and to calculate the number of relatives that need to be tested. The probability of carrying a path-var/mutation in a first-degree relative of a known mutation carrier (following predictive testing) is 50%. The probability of carrying a path-var/mutation in a second-degree relative of a known mutation carrier (following predictive testing) is 25%. The number of unaffected female relative path var carriers identified through cascade testing is calculated to be 1.41 per index path var carrier with BC in the UK and 1.46 per index path var carrier with BC in the USA. Male first-degree relatives were tested to inform the need to test second-degree relatives but they were not followed in the model. Long-term outcomes-&-costs were only modelled for females.

© 2019 American Medical Association. All rights reserved.

eTable 4. Summary of Medical Costs Used in the Model (2016 Prices) and Explanation

Item	UK (£)	US ($)	Source
Cost of genetic testing	175	330	[32,33]
Cost of counselling (per session)	20	40	[34-37]
Cost of RRSO (and HRT and osteoporosis prevention)	3,618	8,476	[38-41]
Cost of ovarian cancer diagnosis and initial treatment	14,268	133,121	[38,40,42]
Yearly cost of ovarian cancer treatment and follow-up: years 1-2	5,433	14,635	[38,40,42,43]
Yearly cost of ovarian cancer treatment and follow-up: years 3-5	5,090	14,635	[38,40,42,43]
Terminal care cost with ovarian cancer	16,452	93,005	[40,44]
Cost of breast cancer screening general	417	1,596	[38,45,46]
Cost of breast cancer screening mutation carriers	5,094	34,896	[38,40,46,47]
Cost of RRM (and reconstruction and complications)	7,421	22,110	[38,40,48-51]
Cost of CPM (and reconstruction and complications)	5,545	20,426	[38,40,51,52]
Cost of chemoprevention	137	4,496	[39,40]
Cost of breast cancer diagnosis and initial treatment (Sporadic, *PALB2*)	19,663	90,040	[38,40,53,54]
Cost of breast cancer diagnosis and initial treatment (*BRCA1/BRCA2*)	17,920	83,633	[38,40,53,54]
Yearly cost of breast cancer follow-up and adjuvant treatment: years 1-5 (Sporadic)	1,436	8,048	[38-40,45,53-55]
Yearly cost of breast cancer follow-up and adjuvant treatment: years 1-5 (*BRCA1/BRCA2*)	1,458	8,048	[38-40,45,53-55]
Yearly cost of breast cancer follow-up and adjuvant treatment: years 1-5 (*PALB2*)	1,438	8,048	[38-40,45,53-56]
Terminal care cost with breast cancer	16,452	68,022	[40,44]
Cost of fatal CHD	3,387	23,934	[38,44,57]
Cost of excess CHD	3,425	196,477	[26,58-62]

BNF – British National Formulary, CPM – contralateral prophylactic mastectomy, GCaPPS – Genetics Cancer Prediction through Population Screening study, HRT – hormone replacement therapy, NHS – National Health Service, NICE – National Institute for Health and Clinical

© 2019 American Medical Association. All rights reserved.

Excellence, PSSRU – Personal Social Services Research Unit, RRSO – risk-reducing salpingo-oophorectomy, RRM – risk-reducing mastectomy. Model costs are estimated at 2016 prices

Explanations:

All costs are adjusted for 2016 price index. Costs were converted wherever needed using the Hospital and Community Health-Service-Index.[63] Costs of breast cancer (BC), ovarian cancer (OC) and excess coronary heart disease (CHD) are included. In line with NICE recommendations, future healthcare costs not associated with BC, OC, or CHD were not considered.[64]

Cost of genetic testing/counselling

The cost of *BRCA1/BRCA2/PALB2* testing is based on testing costs for these genes in the PROMISE research programme as well as confirmatory testing costs in an accredited national genetics laboratory for those testing positive. The UK national unit cost assumed for genetic counselling is £44 per hour of client contact from PSSRU Unit costs of Health and Social Care 2010.[34,35,65] The US cost estimates are obtained from Schwartz et al 2014[36]. All costs are adjusted for the 2016 price index. We assume/cost for 20 minutes of administrator time, 20 minutes of counsellor preparation and 20 min of counselling time (total 40 minutes of counsellor time)[36] for each counselling appointment. In the analysis we include costs for (a) pre-test counselling for all patients, (b) post-test counselling for path-vars and VUS, and (c) also for repeat counselling for VUS which get reclassified as pathogenic subsequently.

RRSO costs

The UK RRSO costs are obtained from NHS reference costs[38], and the US costs are from Grann 2011[40] inflated using the medical component of the US consumer price index to 2016 US$. Costs of HRT for the UK are taken from BNF [39] and for the US from William-Frame 2009.[41] Costs assume HRT is given from average age of RRSO to the average age of menopause (51 years). These costs are calculated for the 80% assumed to be compliant with HRT. Costs include the cost of three follow up DEXA scans for monitoring bone health and calcium and vitamin-D3 for additional osteo-protection.

© 2019 American Medical Association. All rights reserved.

RRM and CPM costs

The UK RRM and CPM costs are obtained from NHS reference costs [38], and the US costs are from Grann 2011[40] inflated using the medical component of the US consumer price index to 2016 US$.

Reconstruction rates of around 91% have been reported after RRM.[50] Costs for the UK are derived from NHS reference costs (code JA33Z).[38] Bilateral prophylactic mastectomy costs for the USA is $20, 827 (2016 price) to include reconstructive surgery.[51] For risk reducing bilateral prophylactic mastectomy (RRM) and reconstruction we assume a 26.2% minor complication rate and 5.6% major complication rate,[51] additional costs for which have been included for both minor and major complications.[51]

Reconstruction rate after contralateral prophylactic mastectomy (CPM) is 90%.[52] Complication rates for contralateral mastectomy are higher than unilateral mastectomy and the major complication rate with reconstruction is higher than without reconstruction. The complication rate for contralateral mastectomy without reconstruction is 42.9% (40.9% minor and 2% major)[52] and the complication rate for contralateral mastectomy and reconstruction is 41.6% (27.7% minor and 13.9% major).[52] Minor complications are costed at an additional cost of $822 (US) and £278 (UK) and major complications at $7492 (US) and £2535 (UK) (2016 prices).[51] All costs are adjusted for 2016 price index. UK costs were converted wherever needed using the Hospital and Community Health-Service-Index.[63]

Costs of ovarian cancer

We assume that the costs of ovarian cancer diagnosis include a pelvic examination, ultrasound scan, CA125 test, CT scan, percutaneous biopsy, and peritoneal cytology. The costs of ovarian cancer treatment include the reference cost for a lower and upper genital tract very complex major procedure and administration of chemotherapy based on 6 cycles of carboplatin and paclitaxel treatment. It is assumed that in the first and second years treated survivors would have a further three consultant visits, a CT scan and four CA125 tests each year. In the third to fifth years post-surgery it is assumed that survivors would have two consultant visits and two CA125 tests.

© 2019 American Medical Association. All rights reserved.

Costs for ovarian cancer diagnosis and treatment in the UK are derived from national reference costs and a recent ovarian cancer guideline developed by NICE[38,42]. Annual costs of ovarian cancer treatment in the US are taken from Grann et al 2011[40] and inflated using the medical component of the US consumer price index to 2016 US$. We include the costs of treatment of recurrence, taken from Cancer Research UK[43] and Grann 2011.[40]

The costs of ovarian cancer terminal care are derived from end-of-life costs for cancer patients based on a report from the National Audit office UK [44]. For the US the terminal care costs for ovarian cancer are obtained from Grann 2011 [40], inflated using the medical component of the US consumer price index to 2016 US$. In line with NICE recommendations future healthcare costs not associated with ovarian cancer are not considered [64].

Costs of breast cancer

In the general population, 10% breast cancer is non-invasive DCIS and 90% is invasive. 95% of invasive breast cancer is early and locally advanced (stage 1-3), and 5% of invasive breast cancer is advanced breast cancer (stage 4).[55] In *BRCA1/2* carriers, 20% of cancers are DCIS and 80% invasive.[66,67] Stage distribution in *PALB2* carriers is assumed to be the same as in the general population, owing to a lack of robust *PALB2* specific data.

Annual breast cancer treatment costs in the USA are obtained from Grann et al 2011,[40] and inflated using the medical component of the USA consumer price index to 2016 US$.

70% of invasive breast cancers are ER-positive,[54,68] among which 49% are premenopausal. 15% of early/locally advanced breast cancers and 25% of advanced breast cancers are HER2-positive. 27% *BRCA1* and 67% *BRCA2* breast cancers are ER-positive; 5% *BRCA1* and 14% *BRCA2* breast cancers are HER2-positive.[69-74] 74% of *PALB2* breast cancers are ER-positive.[56] All costs are adjusted for *BRCA1/BRCA2/PALB2* breast cancers for differences in stage at presentation, the proportion of being non-invasive, and the proportion of being ER-positive or HER2-positive.

© 2019 American Medical Association. All rights reserved.

Diagnosis costs: Whether suspected at breast screening or through presentation to the GP, diagnosis in the breast clinic is made by triple assessment (clinical assessment, mammography, and ultrasound imaging with core biopsy and/or fine needle aspiration cytology).[54] Clinical examination and mammography costs are from the paper by Robertson C et al.[45] Breast ultrasound and biopsy costs are obtained from NHS reference costs.[38] For all patients presented with suspected advanced breast cancer, MRI should be offered to assess for bone metastases.[68]

Sentinel lymph node biopsy (SLNB) costs: SLNB is used for staging axilla for early invasive breast cancer and no evidence of lymph node involvement on ultrasound or a negative ultrasound-guided needle biopsy (73% of early and locally advanced invasive cancers). The SLNB costs are obtained from NHS reference costs including sentinel lymph node scan and unilateral intermediate breast procedures.[38]

Pretreatment axilla ultrasound costs: Pretreatment ultrasound evaluation of the axilla should be performed for all patients being investigated for early invasive breast cancer and, if morphologically abnormal lymph nodes are identified, ultrasound-guided needle sampling should be offered.[54] The commissioning cost of pre-treatment ultrasound evaluation of the breast and axilla is the same as that of the breast only.[55] The costing model considers the cost of ultrasound-guided needle sampling only, obtained from NHS reference costs.[38]

Axillary lymph node dissection (ALND) costs: ALNB is undertaken for lymph node positive cancers (~31% early and locally advanced invasive cancers - NICE guideline and BCCOM project;[54,55,75] 30% node positive for BRCA1/2 breast cancer- familial breast cancer screening studies, breast cancer case series and Early Breast Cancer Trialists' Collaborative Group data).[66,69-71,76]Cost of ALND is assumed to be 25% of the cost of breast surgery as per NICE guideline development group recommendation.[55]

Breast surgery costs include costs of breast conserving surgery (assumed for all non-invasive cancers, and 75% of early/locally advanced invasive cancers) and costs of mastectomy (for 25% early/locally advanced and all advanced cancers). Reconstruction rates following mastectomy are reported to be 34% in the UK[77] and 55% in the US.[52] The complication rate following mastectomy alone is 21.5%

© 2019 American Medical Association. All rights reserved.

(19.5% minor and 2% major)[52] and complication rate following mastectomy and reconstruction is 28.6% (24.5% minor & 4.1% major).[52] Costs are obtained from the national NHS reference costs [38].

Chemotherapy and radiotherapy costs: Invasive breast cancers who are not at low risk [75,78,79] receive adjuvant treatment in line with NICE guidelines. Costs include radiotherapy costs for 60% of early invasive/locally advanced, radiotherapy and chemotherapy costs for 40% early invasive/locally advanced, and chemotherapy for all advanced cancers. Radiotherapy costs include planning and 40Gy in 15 fractions over 3 weeks [54] or palliative treatment, taken from national NHS reference costs [38]. Chemotherapy costs based on polychemotherapy [80], include administration costs, costs of 1st and 2nd line therapy and toxicity from NICE guidelines [55,68].

Endocrine therapy costs: As per NICE guidelines[54,55] , ER-positive invasive breast cancers receive Tamoxifen 20mg/day (premenopausal) or Anastrazole 1mg/day (postmenopausal). 70% of invasive breast cancers are ER-positive [54,68], among which 49% are premenopausal. We assume the length of endocrine therapy is 5 years. The drug costs are obtained from the BNF [39]. ER testing costs are obtained from a local NHS trust and included for all invasive breast cancers.

Target therapy costs: HER2-positive breast cancer patients can be given at 3-week intervals for 1 year or until disease recurrence as per NICE guidelines. Breast cancer patients with positive HER2 are eligible for treatment with trastuzumab [54,68]. 10% of the eligible patients are intolerant of trastuzumab. Among women suitable for this treatment, 80% receive trastuzumab [55]. HER2 testing costs are obtained from a local NHS trust and included for all invasive breast cancers. The trastuzumab cost per patient including administration of treatment and cardiac monitoring is £15080, obtained from NICE costing report [55].

Follow up costs: Breast cancer patients are offered mammographic surveillance and clinical follow-up, with the screening cost of £141.45 per women in 2011[45]. We assume patients are followed up

© 2019 American Medical Association. All rights reserved.

every four months in the first two years, every six months from the third to the fifth year, and every year from the sixth to the tenth year.

Bisphosphonate costs: Bisphosphonates is considered to be offered to patients newly diagnosed with bone metastases, to prevent skeletal-related events and reduce pain [68]. 74% patients with advanced breast cancer will develop bone metastases and 65% patients with bone metastases are offered bisphosphonates[55,81]. Bisphosphonates that are currently offered include oral sodium clodronate, ibandronic acid, zoledronic acid, and pamidronate. The proportions of patients receiving the four drugs are 20%, 30%, 25%, and 25% respectively. The annual costs including administration for the four drugs are £1971, £2541.96, £3208, and £3208 respectively, obtained from NICE costing report [55]. We assume the average length of bisphosphonates treatment is 2.7 years, which is the life expectancy of advanced breast cancers based on one-year survival rate (63.2%) [82].

Recurrence costs: For non-invasive breast cancers, the non-invasive and invasive relapse rates are both 12.5%. 35% of early and locally advanced invasive breast cancers progress to advanced disease [55]. The recurrence rates for early and locally advanced breast cancer are 15.9% for node-positive [83] and 11% for node-negative disease [84]. Weighted for 31% node positive and 69% node negative, the composite recurrence rate for early and locally advanced breast cancer is 12.5%. The recurrence rate for the advanced disease is 66% (34% relapse-free five-year survival) [85].

Terminal care costs: The costs of terminal care for breast cancer are derived from end-of-life costs for cancer patients based on a report from the National Audit office UK [44]. For the US the terminal care costs for breast cancer are obtained from Grann 2011 [40], inflated using the medical component of the US consumer price index to 2016 US$. In line with NICE recommendations future healthcare costs not associated with breast cancer ware not considered [64].

Cost of breast cancer screening

© 2019 American Medical Association. All rights reserved.

For non-carriers, we assume routine triennial mammography between 50-70 years as per UK NHS breast cancer screening programme[86] (seven mammograms on average). Breast screening in the US assumes mammography every two years starting at 50 years.[46]

For *BRCA1/BRCA2/PALB2* mutation carriers, we assume annual mammogram from 40-69 years and annual MRI from 30-49 years as per NICE guidelines for familial breast cancer[47] (30 mammograms and 20 MRIs on average). For the US, it is based on annual mammography and MRI starting at 30 years, and annual mammography only from age 50 years.[46]

Cost of chemoprevention

BRCA1/BRCA2/PALB2 mutation carriers are offered Tamoxifen (premenopausal) or Raloxifene (postmenopausal) for 5 years[47,87] to reduce breast cancer risk. The drug costs are obtained from BNF (UK)[39] and Grann 2011.[40] 16.3% uptake is assumed for chemoprevention.[25]

Cost of CHD

Cost of excess CHD: British Heart Foundation statistics reports costs per capita across four Commissioning Regions in England (London, Midlands and East, North and South) [59].

The costs of CHD and stroke are averaged across the four regions. The prevalence of CHD is estimated at 12.0% in the UK[59] and 11.7% in the USA[60] with the onset of CHD estimated at 55 years of age.[26,58]

The yearly cost of CHD in the UK is obtained by dividing the per capita cost by the population prevalence of CHD.[59] Using the report published by the American Heart Association,[61] the total cost of CHD, CHF and stroke were divided by the population with CHD[60,62] giving the yearly cost of CHD in the USA. This yearly cost is multiplied by the number of years between onset of CHD and average life expectancy to provide the cost attributed to excess CHD.

Cost of fatal CHD: This is costed on the basis of a fatal myocardial infarction using NHS reference costs.[38] USA costs are obtained from Afana et al 2015,[57] inflated using the medical component of the US consumer price index to 2016 US$.

© 2019 American Medical Association. All rights reserved.

In line with NICE recommendations, future healthcare costs not associated with BC, OC, or CHD were not considered.[64]

© 2019 American Medical Association. All rights reserved.

eMethods 1. Examination of Productivity Loss

The retirement ages for females are 65 in the UK and 62 in the USA. The female labour force participation rates are 56.77% in the UK and 55.99% in the USA, obtained from the World Bank [88]. The hourly wage rage are obtained from Office for National Statistics UK[89] and Bureau of Labour Statistics USA [90].

We categorised the productivity costs as three subcomponents: 1) temporary disability due to short-term work absences following diagnosis, 2) permanent disability due to reduced working hours following a return to work or workforce departure; and 3) premature mortality due to death before retirement [91], detailed below.

Descriptive statistics for productivity loss in breast and ovarian cancer patients

**Variables**	**Breast cancer**	**Ovarian cancer**
**(1) Temporary disability**		
Percentage of temporary disability cases	94.0%	98%[1]
Average time taken off work following diagnosis (weeks)	44.9	47.22[2]
**(2) Permanent disability**		
Percentage of permanent disability: reduced hours	26%	40%[3]
Reduced hours per week after returning to work (hours)	5.5	5.5
**(3) Premature mortality (before retirement)**		
Percentage of permanent disability: workforce departure	12.9%	30%[3]

The descriptive statistics for productivity loss in breast cancer patients are obtained from Hanly et al. 2012 [91].

[1] We assume 98% ovarian cancer patients have cancer-related short-term work absences after diagnosis.
[2] We assume ovarian cancer patients experience four weeks for surgery, 24 weeks for chemotherapy, and 24 weeks for recurrence treatment with the recurrence rate of 80% [92].
[3] We assume the percentages of permanent disability for ovarian cancer are 40% for reduced working hours and 30% for workforce departure.

We estimated temporary disability as time absent from work multiplied by age-specific gross earnings.

We calculated productivity costs due to permanent disability by applying age-specific gross earnings to the reduction in working hours, or the number of working hours if permanent workforce departure, until retirement age. Regarding productivity loss from premature mortality, we assumed that without cancer, the productive capacity of an individual would continue from the age of diagnosis until age of retirement. We multiplied the projected years of life lost by the age-specific gross earnings for the remainder of the working life to generate monetary estimates.

© 2019 American Medical Association. All rights reserved.

eMethods 2. Estimates for Age of Onset and Survival for Breast and Ovarian Cancers

Our analysis incorporates lifetime risks and long-term consequences providing a lifetime time-horizon. Female lifetables from the Office of National Statistics (UK women)[30] and National Centre for Health Statistics (USA women)[31] were used for life expectancy by 80-years for women not developing OC/BC.

We assumed that the median age for undergoing RRM and RRSO in unaffected path var carriers was 37 and 40 years respectively.[14] We explored 42 years for RRM and 46 years for RRSO in our scenario-analysis.[93] The uptake rates of RRSO and RRM are obtained from established literature.[14,16] OC/BC outcomes were modelled using 10-year survival data. No statistically significant survival difference between *BRCA1/BRCA2* and sporadic BC has been reported.[94,95] For BC, 10-year survival rates = 78.4% (CI: 78.3,78.4).[96] Long-term survival outcomes for *BRCA* and sporadic OC have also recently been reported to be similar.[97] For OC the 10 year survival rate is 34.5% (CI: 33.8,35.3).[98]

BC and OC survival (from diagnosis to death) were modelled using ten-year survival-data. After ten-years, we assumed the probability of death for all patients was same as the general-population. The excess risk of CHD following premenopausal oophorectomy is incorporated in the analysis.[26,99] We incorporated the fact that contralateral BC is associated with a higher risk of dying from BC.[100] We assume no significant long-term survival difference between germline and sporadic breast/ovarian cancers.[94,95,97]

© 2019 American Medical Association. All rights reserved.

eMethods 3. Quality-Adjusted Life-Years (QALYs) and Utility Scores

QALY is a measurement of health-outcomes in economic evaluations recommended by NICE. It equals time spent in the relevant health states multiplied by an appropriate utility-score. Utility-score is an indication of individual preferences for specific health-states where 1=perfect health and 0=death. Utility-score is an adjustment for quality-of-life and QALY adjusts changes in length-of-life by potential alterations in quality-of-life. The utility-scores for early, advanced, recurrent, remittent, and end-stage BC are 0.71, 0.65, 0.45, 0.81, and 0.16 respectively.[53] The utility-scores for early, advanced, recurrent, remittent, and end-stage OC are 0.81, 0.55, 0.50, 0.83, and 0.16 respectively[101]. In addition, women undergoing RRM or RRSO also experience negative health-effects.[102,103] We used utility-scores of 0.88 (SD=0.22) for RRM, 0.95 (SD=0.10) for RRSO, and 0.84 (SD=0.02) for CHD to account for the disutility.[40,104]

eMethods 4. Patient and Public Involvement Statement

The study team has worked closely with patient support groups like *BRCA* Umbrella and Ask Eve. Increasing access to genetic-testing at cancer diagnosis has been highlighted to the team as an important issue affecting women with cancer and *BRCA*-carriers. Patients have indicated the need for access to unselected genetic-testing for BC. This has been highlighted at patient support days organised and attended by team members as well as in personal communication with leading patient stakeholders (e.g. Caroline Presho, *BRCA*-Umbrella). This work justifies relaxing testing guidelines, a key need highlighted by patients'. Patients did not directly input into the design and conduct of this analysis. Patient support groups and charities will be involved in dissemination of these research findings following acceptance for publication.

© 2019 American Medical Association. All rights reserved.

eFigure 1. Tornado Diagram of 1-Way Sensitivity Analysis

eFigure 1a. Tornado diagram – UK payer perspective

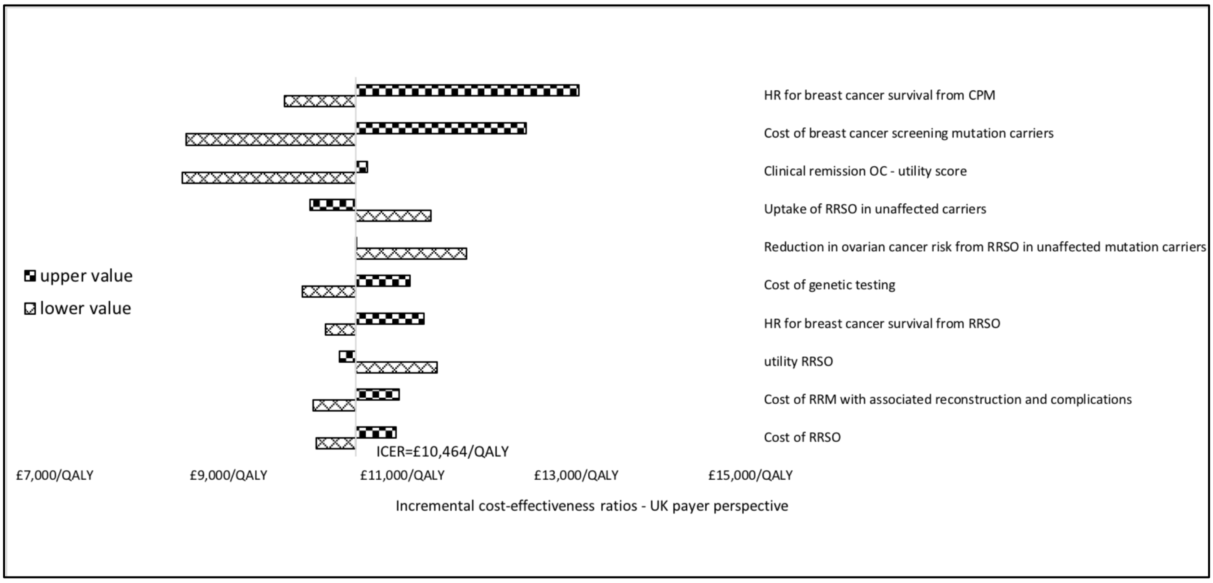

eFigure 1b. Tornado diagram – UK societal perspective

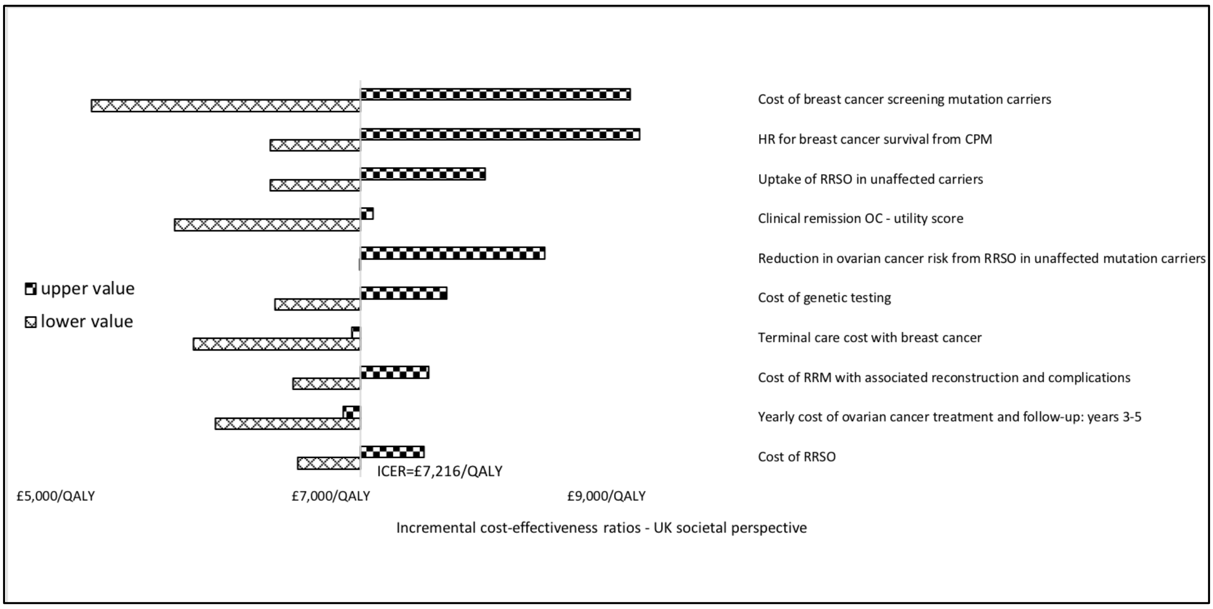

© 2019 American Medical Association. All rights reserved.

eFigure 1c. Tornado diagram – US payer perspective

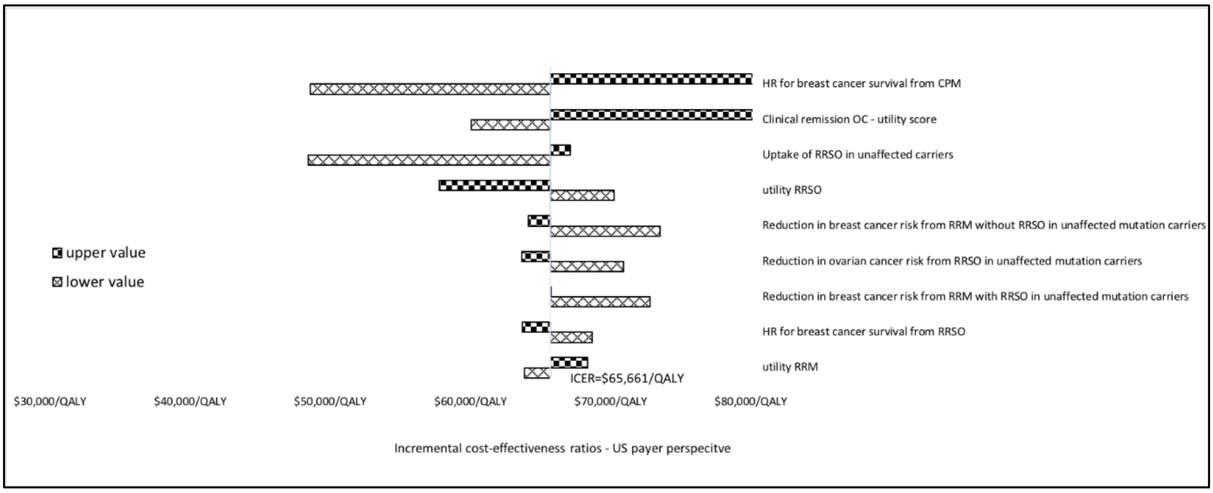

e-Figure-1d. Tornado diagram – US societal perspective

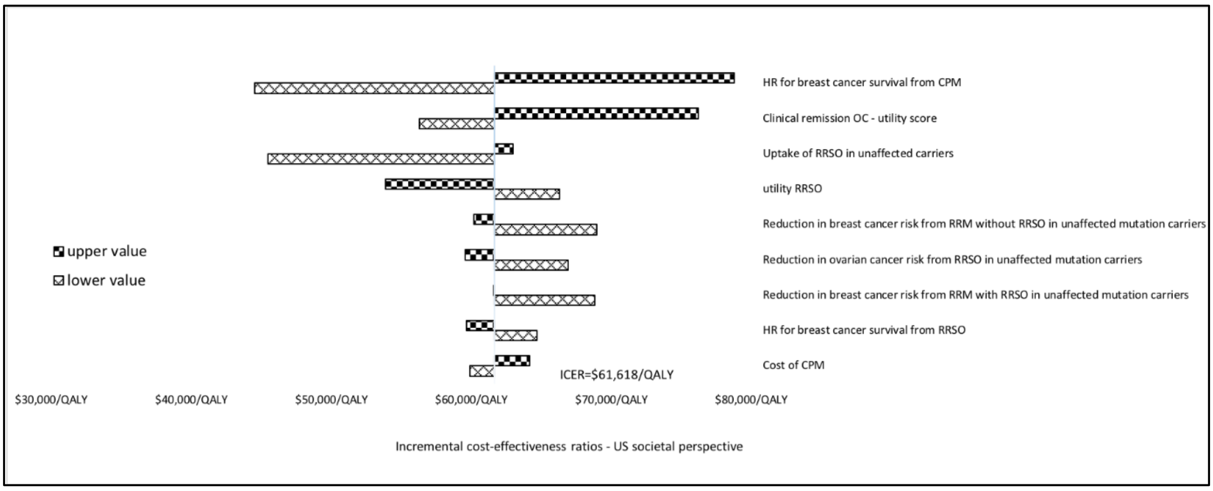

BC – breast cancer, CPM – contralateral prophylactic mastectomy, HR – hazard ratio, ICER-incremental cost-effectiveness ration, OC – ovarian cancer, RRSO – risk-reducing salpingo-oophorectomy, RRM – risk-reducing mastectomy.

One-way sensitivity analysis for all probabilities, costs and utilities in terms of ICER of UK and USA Unselected testing for BRCA1, BRCA2 and PALB2 mutations, compared to a Clinical-criteria / FH-based approach for BRCA1 and BRCA2 testing.

X-axis: Incremental cost-effectiveness ratio (ICER): Cost (£s or $s) per quality adjusted life year (QALY) (discounted).

© 2019 American Medical Association. All rights reserved.

Y-axis: Probability, cost and utility parameters in the model. The model is run at both lower and upper values/limits of the 95% confidence interval or range of all probability parameters described in Table-2; and both lower and upper values/limits of the cost and utility-score parameters given in methods and Appendix 3. Costs are varied by +/- 30%.

'Upper value' represents outcomes for upper limit and 'Lower value' represents outcomes for lower limit of the parameter.

© 2019 American Medical Association. All rights reserved.

eFigure 2. Scatterplots for Incremental Discounted Lifetime Costs and Effects of Unselected Multigene Testing Compared With *BRCA* Testing Based on Family History and Clinical Criteria

eFigure 2a. Incremental discounted costs and QALYs plots - UK payer perspective

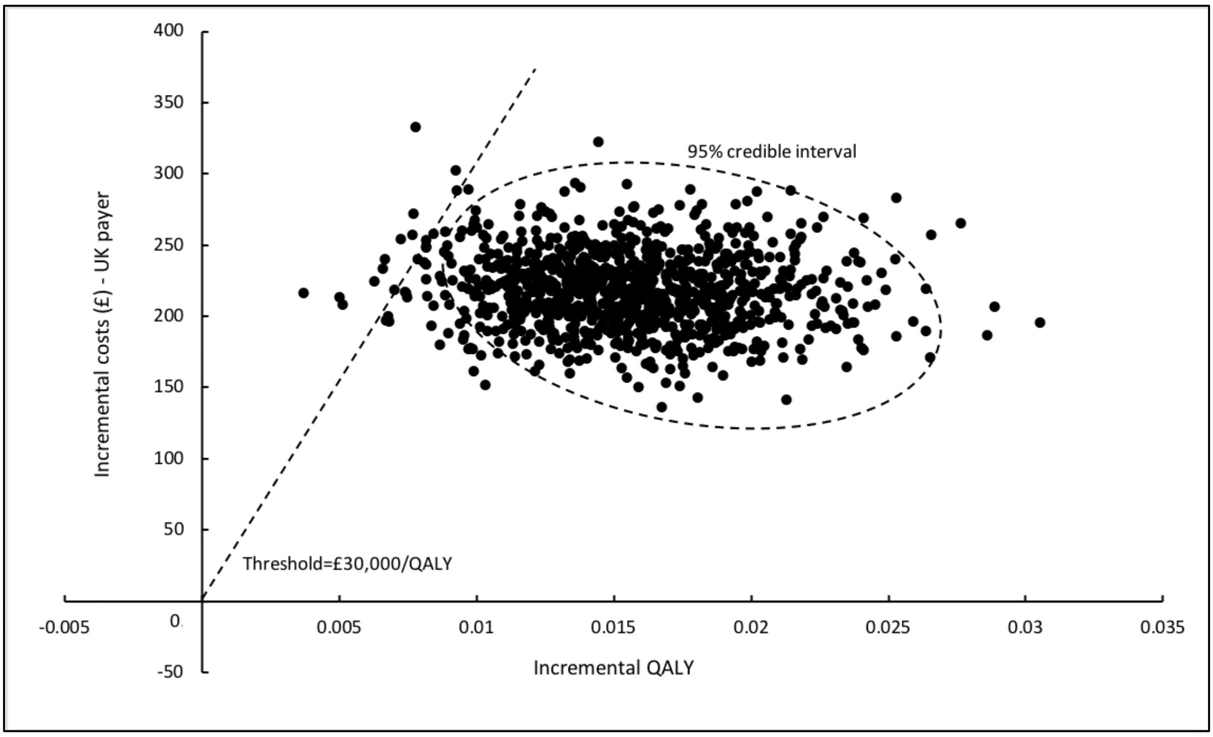

eFigure 2b. Incremental discounted costs and QALYs plots - UK societal perspective

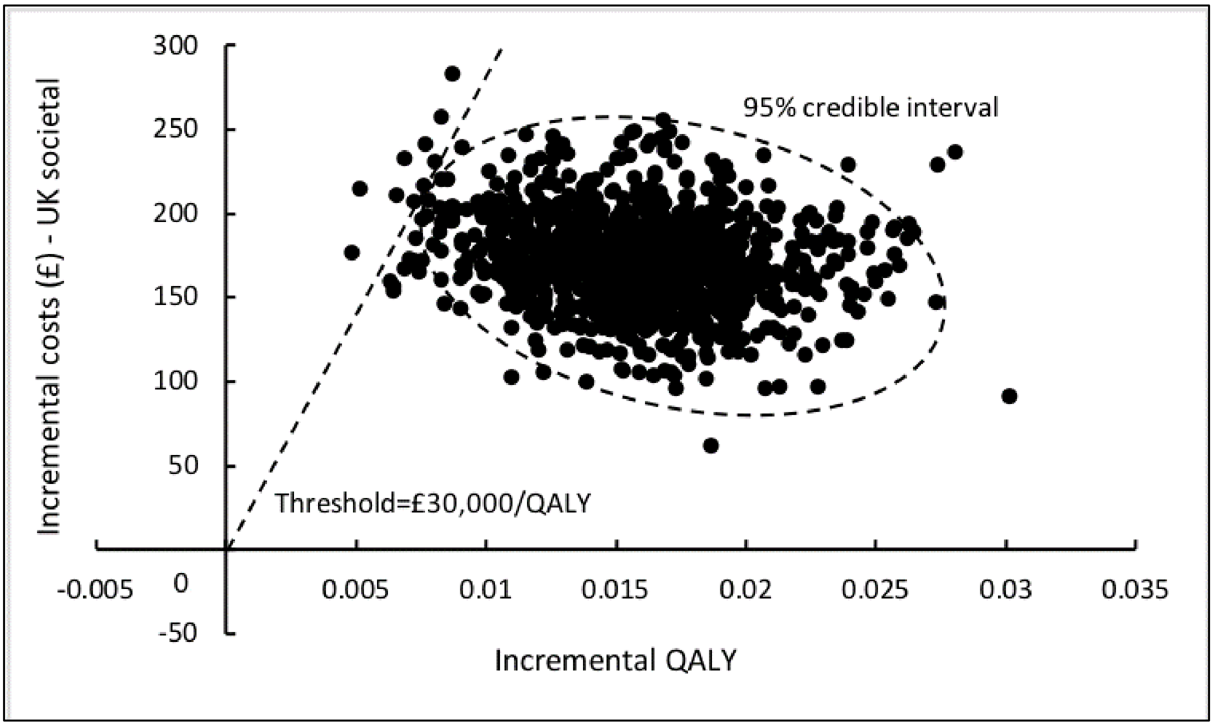

© 2019 American Medical Association. All rights reserved.

eFigure 2c. Incremental discounted costs and QALYs plots – US payer perspective

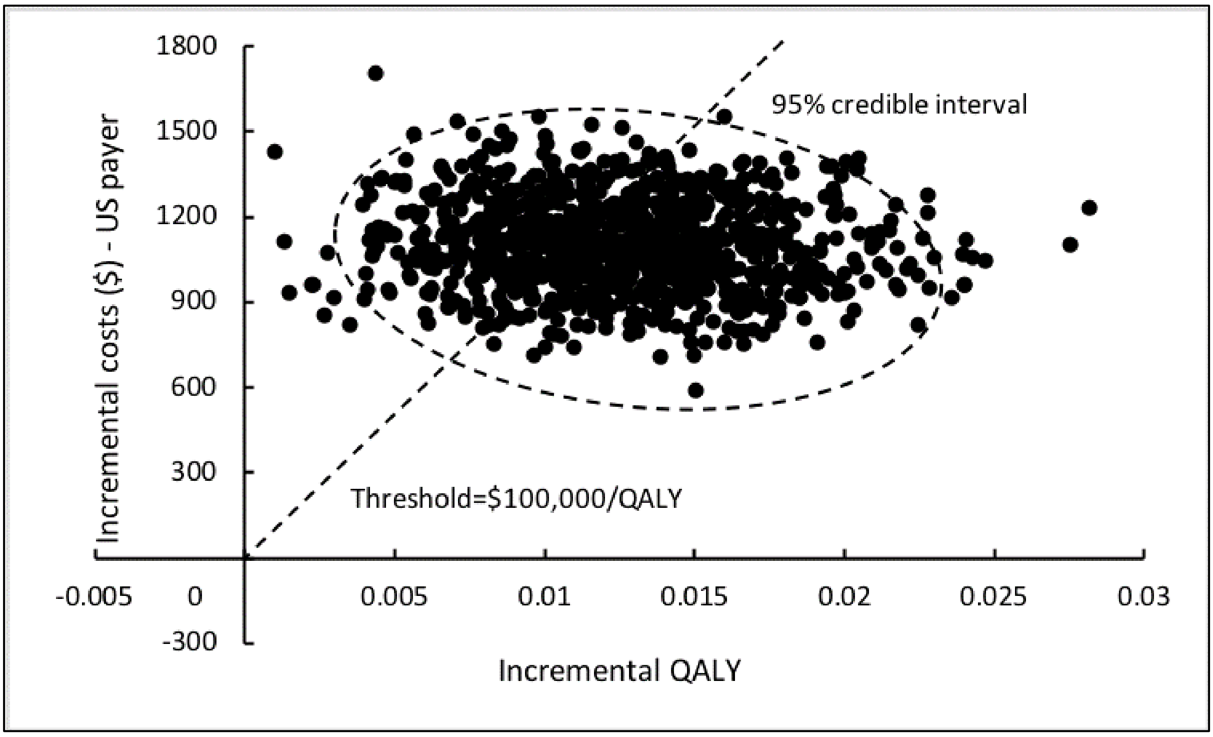

eFigure 2d. Incremental discounted costs and QALYs plots – US societal perspective

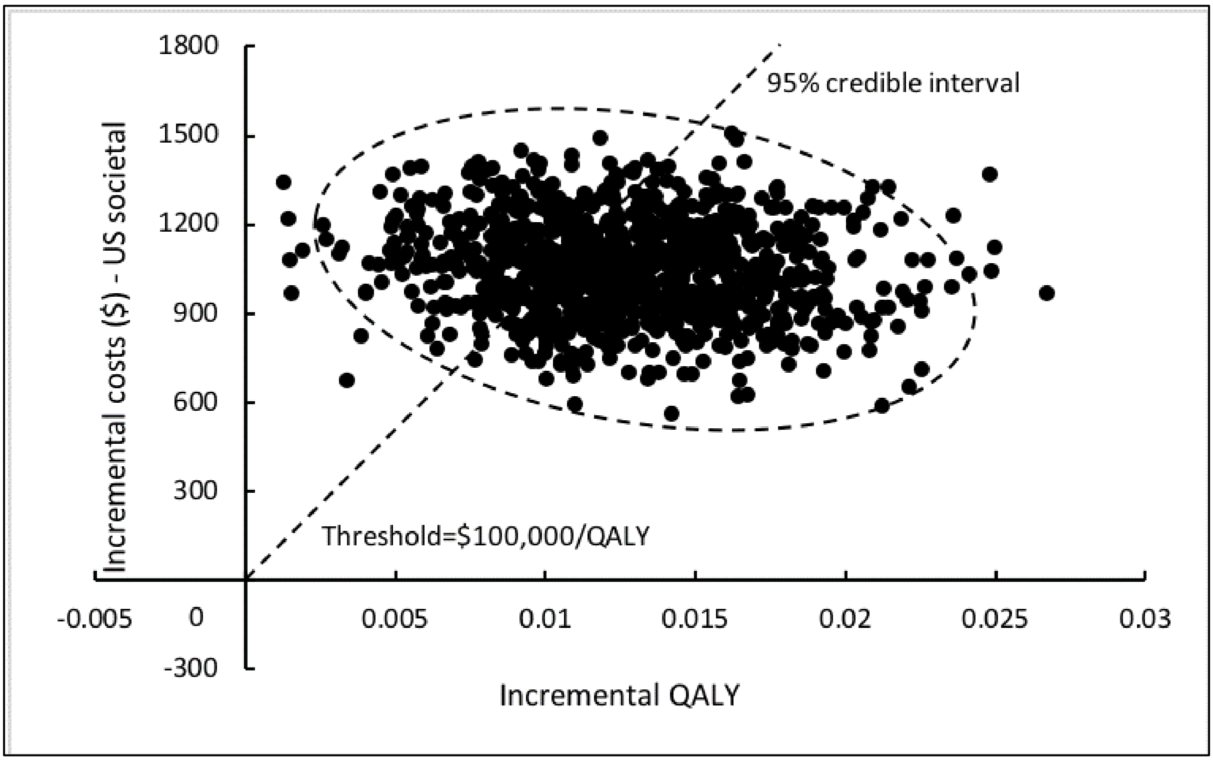

© 2019 American Medical Association. All rights reserved.

The result of each iteration/simulation in the PSA is plotted on the CE plane, The results appear as a “cloud” of possible outcomes. Each point on the scatter plot represents one simulation/bootstrap iteration. All points lie in the North East quadrant of the CE plane, suggesting unselected testing is always more effective. The dotted line represents the willingness to pay threshold, thus enabling interpretation of number of simulations which lie above or below this threshold.

© 2019 American Medical Association. All rights reserved.

eReferences.

1. Cancer Research UK. Breast cancer incidence (invasive) statistics. 2015; http://www.cancerresearchuk.org/health-professional/cancer-statistics/statistics-by-cancer-type/breast-cancer/incidence-invasive#collapseOne. Accessed 14 March, 2018.
2. United States Cancer Statistics. Rate of New Cancers by Age Group, All Races, Female. 2015; https://gis.cdc.gov/Cancer/USCS/DataViz.html. Accessed 19 November, 2018.
3. Evans DG, Astley S, Stavrinos P, et al. Improvement in risk prediction, early detection and prevention of breast cancer in the NHS Breast Screening Programme and family history clinics: a dual cohort study. *Programme Grants for Applied Research.* 2016. https://www.ncbi.nlm.nih.gov/pubmed/27559559.
4. Copson E, Maishman T, Gerty S, et al. Ethnicity and outcome of young breast cancer patients in the United Kingdom: the POSH study. *Br J Cancer.* 2014;110(1):230-241.
5. Brentnall AR, Cuzick J, Buist DSM, Bowles EJA. Long-term Accuracy of Breast Cancer Risk Assessment Combining Classic Risk Factors and Breast Density. *JAMA Oncol.* 2018;4(9):e180174.
6. Buys SS, Sandbach JF, Gammon A, et al. A study of over 35,000 women with breast cancer tested with a 25-gene panel of hereditary cancer genes. *Cancer.* 2017;123(10):1721-1730.
7. Brentnall AR, Cuzick J, Buist DSM, Bowles EJA. Long-term Accuracy of Breast Cancer Risk Assessment Combining Classic Risk Factors and Breast Density. *JAMA Oncol.* 2018:e180174.
8. Copson ER, Maishman TC, Tapper WJ, et al. Germline BRCA mutation and outcome in young-onset breast cancer (POSH): a prospective cohort study. *Lancet Oncol.* 2018;19(2):169-180.
9. Evans DG, Ingham S, Dawe S, et al. Breast cancer risk assessment in 8,824 women attending a family history evaluation and screening programme. *Familial cancer.* 2014;13(2):189-196.
10. National Institute for Health and Care Excellence. *Familial breast cancer: classification, care and managing breast cancer and related risks in people with a family history of breast cancer.* 2013.
11. Slavin TP, Maxwell KN, Lilyquist J, et al. The contribution of pathogenic variants in breast cancer susceptibility genes to familial breast cancer risk. *NPJ Breast Cancer.* 2017;3:22.
12. van Marcke C, Collard A, Vikkula M, Duhoux FP. Prevalence of pathogenic variants and variants of unknown significance in patients at high risk of breast cancer: A systematic review and meta-analysis of gene-panel data. *Crit Rev Oncol Hematol.* 2018;132:138-144.
13. Mersch J, Brown N, Pirzadeh-Miller S, et al. Prevalence of Variant Reclassification Following Hereditary Cancer Genetic Testing. *JAMA.* 2018;320(12):1266-1274.
14. Evans DG, Lalloo F, Ashcroft L, et al. Uptake of risk-reducing surgery in unaffected women at high risk of breast and ovarian cancer is risk, age, and time dependent. *Cancer Epidemiol Biomarkers Prev.* 2009;18(8):2318-2324.
15. Evans DG, Ingham SL, Baildam A, et al. Contralateral mastectomy improves survival in women with BRCA1/2-associated breast cancer. *Breast cancer research and treatment.* 2013;140(1):135-142.
16. Manchanda R, Burnell M, Abdelraheim A, et al. Factors influencing uptake and timing of risk reducing salpingo-oophorectomy in women at risk of familial ovarian cancer: a competing risk time to event analysis. *Bjog.* 2012;119(5):527-536.

© 2019 American Medical Association. All rights reserved.

17. Kauff ND, Domchek SM, Friebel TM, et al. Risk-reducing salpingo-oophorectomy for the prevention of BRCA1- and BRCA2-associated breast and gynecologic cancer: a multicenter, prospective study. *J Clin Oncol.* 2008;26(8):1331-1337.
18. Rebbeck TR, Friebel T, Lynch HT, et al. Bilateral prophylactic mastectomy reduces breast cancer risk in BRCA1 and BRCA2 mutation carriers: the PROSE Study Group. *J Clin Oncol.* 2004;22(6):1055-1062.
19. Rebbeck TR, Kauff ND, Domchek SM. Meta-analysis of risk reduction estimates associated with risk-reducing salpingo-oophorectomy in BRCA1 or BRCA2 mutation carriers. *J Natl Cancer Inst.* 2009;101(2):80-87.
20. Basu NN, Ingham S, Hodson J, et al. Risk of contralateral breast cancer in BRCA1 and BRCA2 mutation carriers: a 30-year semi-prospective analysis. *Fam Cancer.* 2015;14(4):531-538.
21. Finch A, Beiner M, Lubinski J, et al. Salpingo-oophorectomy and the risk of ovarian, fallopian tube, and peritoneal cancers in women with a BRCA1 or BRCA2 Mutation. *JAMA.* 2006;296(2):185-192.
22. Metcalfe K, Lynch HT, Foulkes WD, et al. Effect of Oophorectomy on Survival After Breast Cancer in BRCA1 and BRCA2 Mutation Carriers. *JAMA Oncol.* 2015;1(3):306-313.
23. Read MD, Edey KA, Hapeshi J, Foy C. Compliance with estrogen hormone replacement therapy after oophorectomy: a prospective study. *Menopause international.* 2010;16(2):60-64.
24. Cuzick J, Sestak I, Cawthorn S, et al. Tamoxifen for prevention of breast cancer: extended long-term follow-up of the IBIS-I breast cancer prevention trial. *Lancet Oncol.* 2015;16(1):67-75.
25. Smith SG, Sestak I, Forster A, et al. Factors affecting uptake and adherence to breast cancer chemoprevention: a systematic review and meta-analysis. *Ann Oncol.* 2016;27(4):575-590.
26. Parker WH, Feskanich D, Broder MS, et al. Long-term mortality associated with oophorectomy compared with ovarian conservation in the nurses' health study. *Obstet Gynecol.* 2013;121(4):709-716.
27. Hopper JL, Chenevix-Trench G, Jolley DJ, et al. Design and analysis issues in a population-based, case-control-family study of the genetic epidemiology of breast cancer and the Co-operative Family Registry for Breast Cancer Studies (CFRBCS). *J Natl Cancer Inst Monogr.* 1999(26):95-100.
28. Office for National Statistics. Cohort Fertility: England and Wales. 2012; https://www.ons.gov.uk/peoplepopulationandcommunity/birthsdeathsandmarriages/conceptionandfertilityrates/datasets/cohortfertilityenglandandwales. Accessed 20 March, 2018.
29. National Center for Health Statistics. Cohort Fertility Tables. 2006; https://www.cdc.gov/nchs/nvss/cohort_fertility_tables.htm. Accessed 20 Nov, 2018.
30. Office of National Statistics. Lifetable for females in the UK. 2011; Office for National Statistics licensed under the Open Government Licence v.1.0. . Available at: http://www.ons.gov.uk/ons/taxonomy/index.html?nscl=Interim+Life+Tables. Accessed 01 June 2012, 2012.
31. Arias E, Heron M, Xu J. United States Life Tables, 2014. *National vital statistics reports : from the Centers for Disease Control and Prevention, National Center for Health Statistics, National Vital Statistics System.* 2017;66(4):1-64.
32. PROMISE FS. Predicting risk of ovarian malignancy improved screening and early detection: Feasibility study 2017: DOI 10.1186/ISRCTN54246466. http://www.isrctn.com/ISRCTN54246466. Accessed 20/08/2017.

© 2019 American Medical Association. All rights reserved.

33. Color Genomics. https://www.color.com/. Accessed 31/07/2018.
34. Manchanda R, Loggenberg K, Sanderson S, et al. Population testing for cancer predisposing BRCA1/BRCA2 mutations in the Ashkenazi-Jewish community: a randomized controlled trial. *J Natl Cancer Inst.* 2015;107(1):379.
35. Curtis L. *Unit Costs of Health and Social Care 2010.* Canterbury, Kent: Personal Social Services Research Unit (PSSRU);2010.
36. Schwartz MD, Valdimarsdottir HB, Peshkin BN, et al. Randomized noninferiority trial of telephone versus in-person genetic counseling for hereditary breast and ovarian cancer. *J Clin Oncol.* 2014;32(7):618-626.
37. Eggington JM, Bowles KR, Moyes K, et al. A comprehensive laboratory-based program for classification of variants of uncertain significance in hereditary cancer genes. *Clin Genet.* 2014;86(3):229-237.
38. Department of Health. *NHS reference costs 2015 to 2016.* 2016.
39. British National Formulary. *British National Formulary.* London: BMJ Group and Pharmaceutical Press (Royal Pharmaceutical Society of Great Britain); 2018.
40. Grann VR, Patel PR, Jacobson JS, et al. Comparative effectiveness of screening and prevention strategies among BRCA1/2-affected mutation carriers. *Breast Cancer Res Treat.* 2011;125(3):837-847.
41. Williams-Frame A, Carpenter JS. Costs of hormonal and nonhormonal prescription medications for hot flashes. *Womens Health (Lond).* 2009;5(5):497-502.
42. National Institute for Health and Care Excellence (NICE). *Ovarian cancer: the recognition and initial management of ovarian cancer.* London: National Institute for Health and Clinical Excellence (NICE);2011.
43. Incisive Health and Cancer Research UK. *Saving lives, averting costs. An analysis of the financial implications of achieving earlier diagnosis of colorectal, lung and ovarian cancer.* 2014.
44. National Audit Office. *End of life care.* London: National Audit Office (NAO), House of Commons;2008.
45. Robertson C, Arcot Ragupathy SK, Boachie C, et al. The clinical effectiveness and cost-effectiveness of different surveillance mammography regimens after the treatment for primary breast cancer: systematic reviews registry database analyses and economic evaluation. *Health Technol Assess.* 2011;15(34):v-vi, 1-322.
46. CDC. Breast Cancer Screening Guidelines for Women. 2016; https://www.cdc.gov/cancer/breast/pdf/breastcancerscreeningguidelines.pdf.
47. National Institute for Health and Care Excellence. *Familial breast cancer: Classification and care of people at risk of familial breast cancer and management of breast cancer and related risks in people with a family history of breast cancer.* London, UK: National Institute for Health and Care Excellence;2013.
48. Contant CM, Menke-Pluijmers MB, Seynaeve C, et al. Clinical experience of prophylactic mastectomy followed by immediate breast reconstruction in women at hereditary risk of breast cancer (HB(O)C) or a proven BRCA1 and BRCA2 germ-line mutation. *Eur J Surg Oncol.* 2002;28(6):627-632.
49. Nelson HD, Fu R, Goddard K, et al. U.S. Preventive Services Task Force Evidence Syntheses, formerly Systematic Evidence Reviews. In: *Risk Assessment, Genetic Counseling, and Genetic Testing for BRCA-Related Cancer: Systematic Review to Update the U.S. Preventive Services Task Force Recommendation.* Rockville (MD): Agency for Healthcare Research and Quality (US); 2013.
50. Neuburger J, Macneill F, Jeevan R, van der Meulen JH, Cromwell DA. Trends in the use of bilateral mastectomy in England from 2002 to 2011: retrospective analysis of hospital episode statistics. *BMJ Open.* 2013;3(8).

© 2019 American Medical Association. All rights reserved.

51. Del Corral GA, Wes AM, Fischer JP, Serletti JM, Wu LC. Outcomes and Cost Analysis in High-Risk Patients Undergoing Simultaneous Free Flap Breast Reconstruction and Gynecologic Procedures. *Ann Plast Surg.* 2015;75(5):534-538.
52. Miller ME, Czechura T, Martz B, et al. Operative risks associated with contralateral prophylactic mastectomy: a single institution experience. *Ann Surg Oncol.* 2013;20(13):4113-4120.
53. National Institute for Health and Care Excellence (NICE). *Clinical Guideline (CG81) – Advanced breast cancer: diagnosis and treatment.* Cardiff, Wales, UK: National Collaborating Centre for Cancer, National Institute for Health and Clinical Excellence;2009.
54. National Institute for Health and Care Excellence (NICE). *Early and locally advanced breast cancer: diagnosis and treatment.* Cardiff, Wales, UK: National Institute for Health and Clinical Excellence;2009.
55. National Institute for Health and Clinical Excellence. *National costing report: Early and locally advanced breast cancer/Advanced breast cancer.* London, UK: National Institute for Health and Clinical Excellence;2009.
56. Antoniou AC, Casadei S, Heikkinen T, et al. Breast-cancer risk in families with mutations in PALB2. *N Engl J Med.* 2014;371(6):497-506.
57. Afana M, Brinjikji W, Cloft H, Salka S. Hospitalization costs for acute myocardial infarction patients treated with percutaneous coronary intervention in the United States are substantially higher than Medicare payments. *Clin Cardiol.* 2015;38(1):13-19.
58. Who Is at Risk for Coronary Heart Disease? 2016; https://www.nhlbi.nih.gov/health-topics/coronary-heart-disease#Risk-Factors. Accessed 17 March, 2018.
59. Townsend N, Bhatnagar P, Wilkins E. *Cardiovascular disease statistics.* London, UK: British Heart Foundation;2015.
60. CDC. Heart Disease. 2017; https://www.cdc.gov/nchs/fastats/heart-disease.htm. Accessed 17 March, 2018.
61. American Heart Association. Cardiovascular Disease: A Costly Burden for America — Projections through 2035. 2017; http://www.heart.org/idc/groups/heart-public/@wcm/@adv/documents/downloadable/ucm_491543.pdf.
62. Population, total. Internation Database; 2017. http://data.worldbank.org/indicator/SP.POP.TOTL.
63. Curtis L. *Unit Costs of Health and Social Care 2011.* Canterbury, Kent: Personal Social Services Research Unit (PSSRU);2011.
64. National Institute of Health and Clinical Excellence. *Guide to the methods of technology appraisal.* London2013.
65. Manchanda R, Burnell M, Loggenberg K, et al. Cluster-randomised non-inferiority trial comparing DVD-assisted and traditional genetic counselling in systematic population testing for BRCA1/2 mutations. *J Med Genet.* 2016;53(7):472-480.
66. Heijnsdijk EA, Warner E, Gilbert FJ, et al. Differences in natural history between breast cancers in BRCA1 and BRCA2 mutation carriers and effects of MRI screening-MRISC, MARIBS, and Canadian studies combined. *Cancer Epidemiol Biomarkers Prev.* 2012;21(9):1458-1468.
67. Nelson HD, Pappas M, Zakher B, Mitchell JP, Okinaka-Hu L, Fu R. Risk assessment, genetic counseling, and genetic testing for BRCA-related cancer in women: a systematic review to update the U.S. Preventive Services Task Force recommendation. *Ann Intern Med.* 2014;160(4):255-266.

© 2019 American Medical Association. All rights reserved.

68. National Institute for Health and Care Excellence (NICE). *Advanced breast cancer: diagnosis and treatment.* Cardiff, Wales, UK: National Institute for Health and Clinical Excellence;2009.
69. Cortesi L, Turchetti D, Marchi I, et al. Breast cancer screening in women at increased risk according to different family histories: an update of the Modena Study Group experience. *BMC Cancer.* 2006;6:210.
70. Leach MO, Boggis CR, Dixon AK, et al. Screening with magnetic resonance imaging and mammography of a UK population at high familial risk of breast cancer: a prospective multicentre cohort study (MARIBS). *Lancet.* 2005;365(9473):1769-1778.
71. Robson ME, Chappuis PO, Satagopan J, et al. A combined analysis of outcome following breast cancer: differences in survival based on BRCA1/BRCA2 mutation status and administration of adjuvant treatment. *Breast cancer research : BCR.* 2004;6(1):R8-R17.
72. Comen E, Davids M, Kirchhoff T, Hudis C, Offit K, Robson M. Relative contributions of BRCA1 and BRCA2 mutations to "triple-negative" breast cancer in Ashkenazi Women. *Breast Cancer Res Treat.* 2011;129(1):185-190.
73. Tung N, Garber JE, Lincoln A, Domchek SM. Frequency of triple-negative breast cancer in BRCA1 mutation carriers: comparison between common Ashkenazi Jewish and other mutations. *J Clin Oncol.* 2012;30(35):4447-4448.
74. Chappuis PO, Nethercot V, Foulkes WD. Clinico-pathological characteristics of BRCA1- and BRCA2-related breast cancer. *Seminars in surgical oncology.* 2000;18(4):287-295.
75. Bates T, Kearins O, Monypenny I, Lagord C, Lawrence G. Clinical outcome data for symptomatic breast cancer: the Breast Cancer Clinical Outcome Measures (BCCOM) Project. *Br J Cancer.* 2009;101(3):395-402.
76. Effects of chemotherapy and hormonal therapy for early breast cancer on recurrence and 15-year survival: an overview of the randomised trials. *Lancet.* 2005;365(9472):1687-1717.
77. Jeevan R, Mennie JC, Mohanna PN, O'Donoghue JM, Rainsbury RM, Cromwell DA. National trends and regional variation in immediate breast reconstruction rates. *Br J Surg.* 2016;103(9):1147-1156.
78. Blamey RW, Ellis IO, Pinder SE, et al. Survival of invasive breast cancer according to the Nottingham Prognostic Index in cases diagnosed in 1990-1999. *Eur J Cancer.* 2007;43(10):1548-1555.
79. Jonathan G, Robyn D. *Adjuvant! Online: review of evidence concerning its validity, and other considerations relating to its use in the NHS.* Cardiff, Wales, UK: National Institute for Health and Clinical Excellence;2009.
80. Early Breast Cancer Trialists' Collaborative G. Effects of chemotherapy and hormonal therapy for early breast cancer on recurrence and 15-year survival: an overview of the randomised trials. *Lancet.* 2005;365(9472):1687-1717.
81. Kozlow W, Guise TA. Breast cancer metastasis to bone: mechanisms of osteolysis and implications for therapy. *Journal of mammary gland biology and neoplasia.* 2005;10(2):169-180.
82. Breast Cancer (C50), One-Year Age Standardised Net Survival by Stage, Adults (Ages 15-99 Years), England 2014. 2014. https://www.cancerresearchuk.org/health-professional/cancer-statistics/statistics-by-cancer-type/breast-cancer/survival.
83. Wapnir IL, Anderson SJ, Mamounas EP, et al. Prognosis after ipsilateral breast tumor recurrence and locoregional recurrences in five National Surgical Adjuvant Breast and Bowel Project node-positive adjuvant breast cancer trials. *J Clin Oncol.* 2006;24(13):2028-2037.

© 2019 American Medical Association. All rights reserved.

84. Anderson SJ, Wapnir I, Dignam JJ, et al. Prognosis after ipsilateral breast tumor recurrence and locoregional recurrences in patients treated by breast-conserving therapy in five National Surgical Adjuvant Breast and Bowel Project protocols of node-negative breast cancer. *J Clin Oncol.* 2009;27(15):2466-2473.
85. Gennari A, Conte P, Rosso R, Orlandini C, Bruzzi P. Survival of metastatic breast carcinoma patients over a 20-year period: a retrospective analysis based on individual patient data from six consecutive studies. *Cancer.* 2005;104(8):1742-1750.
86. Waldron J. *Breast Screening Programme, England 2008-09.* UK: The NHS Information Centre, for Health and Social Care, Government Statistical Service;2010.
87. Cuzick J, Sestak I, Bonanni B, et al. Selective oestrogen receptor modulators in prevention of breast cancer: an updated meta-analysis of individual participant data. *Lancet.* 2013;381(9880):1827-1834.
88. The World Bank. Labor force participation rate, female (% of female population ages 15+) (modeled ILO estimate). 2018; https://data.worldbank.org/indicator/SL.TLF.CACT.FE.ZS. Accessed 7 Nov, 2018.
89. Office for National Statistics. Employee earnings in the UK: 2018. 2018; https://www.ons.gov.uk/employmentandlabourmarket/peopleinwork/earningsandworkinghours/bulletins/annualsurveyofhoursandearnings/2018. Accessed 7 Nov, 2018.
90. Bureau of Labor Statistics. Labor Force Statistics from the Current Population Survey. 2016; https://www.bls.gov/cps/earnings.htm#demographics. Accessed 7 Nov, 2018.
91. Hanly P, Timmons A, Walsh PM, Sharp L. Breast and prostate cancer productivity costs: a comparison of the human capital approach and the friction cost approach. *Value Health.* 2012;15(3):429-436.
92. National Ovarian Cancer Coalition. Ovarian Cancer Recurrence: Discussion With an Expert. 2016; http://ovarian.org/component/content/article/33/385. Accessed 7 Nov, 2018.
93. Metcalfe K, Eisen A, Senter L, et al. International trends in the uptake of cancer risk reduction strategies in women with a BRCA1 or BRCA2 mutation. *Br J Cancer.* 2019.
94. Bordeleau L, Panchal S, Goodwin P. Prognosis of BRCA-associated breast cancer: a summary of evidence. *Breast Cancer Res Treat.* 2010;119(1):13-24.
95. Rennert G, Bisland-Naggan S, Barnett-Griness O, et al. Clinical outcomes of breast cancer in carriers of BRCA1 and BRCA2 mutations. *N Engl J Med.* 2007;357(2):115-123.
96. Cancer Research UK. Breast Cancer Survival Statistics. One-, five-, and ten-year survival. 2011; http://www.cancerresearchuk.org/cancer-info/cancerstats/types/breast/survival/breast-cancer- survival-statistics:http://www.cancerresearchuk.org/cancer-info/cancerstats/types/breast/survival/breast-cancer-survival-statistics. http://www.cancerresearchuk.org/cancer-info/cancerstats/types/breast/survival/breast-cancer-survival-statistics. Accessed 13 March, 2018.
97. McLaughlin JR, Rosen B, Moody J, et al. Long-term ovarian cancer survival associated with mutation in BRCA1 or BRCA2. *J Natl Cancer Inst.* 2013;105(2):141-148.
98. Cancer Research UK. Ovarian Cancer Survival Statistics. One-, five-, and ten-year survival. 2011; https://www.cancerresearchuk.org/health-professional/cancer-statistics/statistics-by-cancer-type/ovarian-cancer/survival#heading-Zero. Accessed 21 Nov, 2018.

© 2019 American Medical Association. All rights reserved.

99. Parrish HM, Carr CA, Hall DG, King TM. Time interval from castration in premenopausal women to development of excessive coronary atherosclerosis. *Am J Obstet Gynecol.* 1967;99(2):155-162.
100. Vichapat V, Garmo H, Holmqvist M, et al. Tumor stage affects risk and prognosis of contralateral breast cancer: results from a large Swedish-population-based study. *Journal of clinical oncology : official journal of the American Society of Clinical Oncology.* 2012;30(28):3478-3485.
101. Havrilesky LJ, Broadwater G, Davis DM, et al. Determination of quality of life-related utilities for health states relevant to ovarian cancer diagnosis and treatment. *Gynecologic oncology.* 2009;113(2):216-220.
102. Grann VR, Patel P, Bharthuar A, et al. Breast cancer-related preferences among women with and without BRCA mutations. *Breast Cancer Res Treat.* 2010;119(1):177-184.
103. Parker WH, Jacoby V, Shoupe D, Rocca W. Effect of bilateral oophorectomy on women's long-term health. *Women's health (London, England).* 2009;5(5):565-576.
104. van Kempen BJ, Spronk S, Koller MT, et al. Comparative effectiveness and cost-effectiveness of computed tomography screening for coronary artery calcium in asymptomatic individuals. *J Am Coll Cardiol.* 2011;58(16):1690-1701.

© 2019 American Medical Association. All rights reserved.
